# Alternative splicing and genetic variation of mhc-e: implications for rhesus cytomegalovirus-based vaccines

**DOI:** 10.1038/s42003-022-04344-2

**Published:** 2022-12-19

**Authors:** Hayden Brochu, Ruihan Wang, Tammy Tollison, Chul-Woo Pyo, Alexander Thomas, Elizabeth Tseng, Lynn Law, Louis J. Picker, Michael Gale, Daniel E. Geraghty, Xinxia Peng

**Affiliations:** 1 Department of Molecular Biomedical Sciences, North Carolina State University College of Veterinary Medicine, Raleigh, NC 27607 USA; 2 Bioinformatics Graduate Program, North Carolina State University, Raleigh, NC 27695 USA; 3 Clinical Research Division, Fred Hutchinson Cancer Research Center, Seattle, WA 98109 USA; 4 Pacific Biosciences, Menlo Park, CA 94025 USA; 5 Department of Immunology, University of Washington, Seattle, WA USA; 6 Center for Innate Immunity and Immune Diseases, University of Washington, Seattle, WA USA; 7 Vaccine and Gene Therapy Institute, Oregon Health & Science University, Beaverton, OR 97006 USA; 8 Washington National Primate Research Center, University of Washington, Seattle, WA USA; 9 Bioinformatics Research Center, North Carolina State University, Raleigh, NC 27695 USA

**Keywords:** Genome informatics, Genomics, Live attenuated vaccines, Immunogenetics

## Abstract

Rhesus cytomegalovirus (RhCMV)-based vaccination against Simian Immunodeficiency virus (SIV) elicits MHC-E-restricted CD8+ T cells that stringently control SIV infection in ~55% of vaccinated rhesus macaques (RM). However, it is unclear how accurately the RM model reflects *HLA-E* immunobiology in humans. Using long-read sequencing, we identified 16 *Mamu-E* isoforms and all *Mamu-E* splicing junctions were detected among *HLA-E* isoforms in humans. We also obtained the complete *Mamu-E* genomic sequences covering the full coding regions of 59 RM from a RhCMV/SIV vaccine study. The *Mamu-E* gene was duplicated in 32 (54%) of 59 RM. Among four groups of *Mamu-E* alleles: three ~5% divergent full-length allele groups (G1, G2, G2_LTR) and a fourth monomorphic group (G3) with a deletion encompassing the canonical *Mamu-E* exon 6, the presence of G2_LTR alleles was significantly (p = 0.02) associated with the lack of RhCMV/SIV vaccine protection. These genomic resources will facilitate additional *MHC-E* targeted translational research.

## Abstract (web summary)

The alternative splicing and genetic variations across the whole MHC-E locus of Rhesus Macaques (Mamu-E) are analysed, which provides a foundation for more comprehensive research of Mamu-E and informs translational research of CMV-based vaccines.

## Introduction

The major histocompatibility complex (MHC) plays an essential role in host immune regulation. MHC is constitutively expressed in nearly all nucleated cells and harbors significant genomic complexity^1–4^. Assigned with the critical role of distinguishing self from non-self, MHC Class I and II genes contain genetic variations that have been associated with hundreds of autoimmune and infectious diseases in human^5–7^. Rhesus macaques (RMs) have been an important nonhuman primate model for the study of many of these human diseases^8^ and are critical for pre-clinical trial vaccine development for protection against human immunodeficiency virus (HIV) using SIV infection in RMs^9,10^. RMs also serve as vaccination models against SARS-CoV-2^11^, *Mycobacterium tuberculosis*^12,13^, and influenza A virus^14^. Intriguingly, the genetic architecture and polymorphisms of MHC class I and II genes differ significantly among primates^15^, posing a challenge for translational interpretation of non-human primate models in general.

Among primate MHC Class I genes, the *MHC-E* locus is long considered as the most conserved^16,17^ and is believed to exist without duplication in both RM and human^18^. As a non-classical MHC molecule, MHC-E dually functions in innate and adaptive immunity by interacting with T cells in addition to NK cells^19^. This unconventional role of *MHC-E* in T-cell immunity is conserved between humans and RMs^20^. Furthermore, human leukocyte antigen (HLA)-E, the human *MHC-E* ortholog, possesses the ability to present both self- and pathogen-derived sequences^21,22^, and its surface expression can be induced by human cytomegalovirus (hCMV)^23^. Together, these unique characteristics make *MHC-E* a crucial target for ongoing CMV-based vaccine development^24–26^.

In a recent rhesus RhCMV/SIV vaccine study, 55% of RMs were protected from a highly pathogenic strain of SIV^9,10^. It was later shown that this protection was driven by RM MHC-E (Mamu-E)-restricted peptide antigen recognition by CD8^+^ T cells^27,28^. Furthermore, Mamu-E intracellular transport is now known to be necessary for vaccine efficacy and is driven by the genetic architecture of RhCMV^29^. We also recently showed that an Interleukin-15 response signature in whole blood predicts RhCMV/SIV vaccine efficacy^30^, but it is still not clear if *Mamu-E* genetic diversity might also contribute to differences in RhCMV/SIV protection outcome.

Evidence suggests *MHC-E* expression and function may be regulated by alternative splicing. The most recent RefSeq annotations for *HLA-E* and *Mamu-E* contain a single transcript with the canonical MHC Class I exon/intron splicing, originally described by Malissen et al.^31^, and three additional *HLA-E* transcript variants predicted using EST and mRNA support. In contrast, *HLA-G*, a separate MHC Class Ib gene, has seven known transcript variants: four membrane-bound and three secreted in soluble form^32^. *Mamu-AG*, the RM ortholog, also shares this extensive alternative splicing^33^. There is an increasing body of evidence linking soluble (s)HLA with downregulated T cell responses^34^ and a variety of immune disorders^35–37^. These sHLA molecules can result from surface shedding, cleavage by metalloproteinases, or secretion via alternative splicing^38^. While a secreted *sHLA-E* transcript has not yet been documented, there is some support from the western blotting of endothelial cells^39^. A more recent study reported an increase of sHLA-E after Japanese Encephalitis Viral infection but did not determine the source^40^. Overall, however, the documentation of this *HLA-E* alternative splicing is sparse, and nothing to our knowledge has been reported for *Mamu-E*.

Given the extreme genomic complexity of the rhesus MHC region, in this study, we aimed to expand genomic resources for *Mamu-E* using long-read sequencing of RM RNAs and DNAs. We characterized *Mamu-E* and *HLA-E* alternatively spliced transcripts, determined their functional capacities, and examined the extent to which their alternative splicing repertoires are conserved. Separately, we interrogated the genetics of RMs from an RhCMV/SIV vaccine study, identifying for the first time extensive *Mamu-E* gene duplications. Finally, we show the potential of these resources by examining the relationship between the *Mamu-E* spliceosome, genetics, and vaccine-induced immunity in whole blood during the pre-challenge phase of an RhCMV/SIV vaccine study^30^. These resources will provide a foundation for more comprehensive research of *MHC-E* in RMs and inform translational research of CMV-based vaccines for use in humans.

## Results

### The gene expression of *Mamu-E* is regulated by extensive alternative splicing that is conserved among *HLA-E* isoforms

**Fig. 1:**
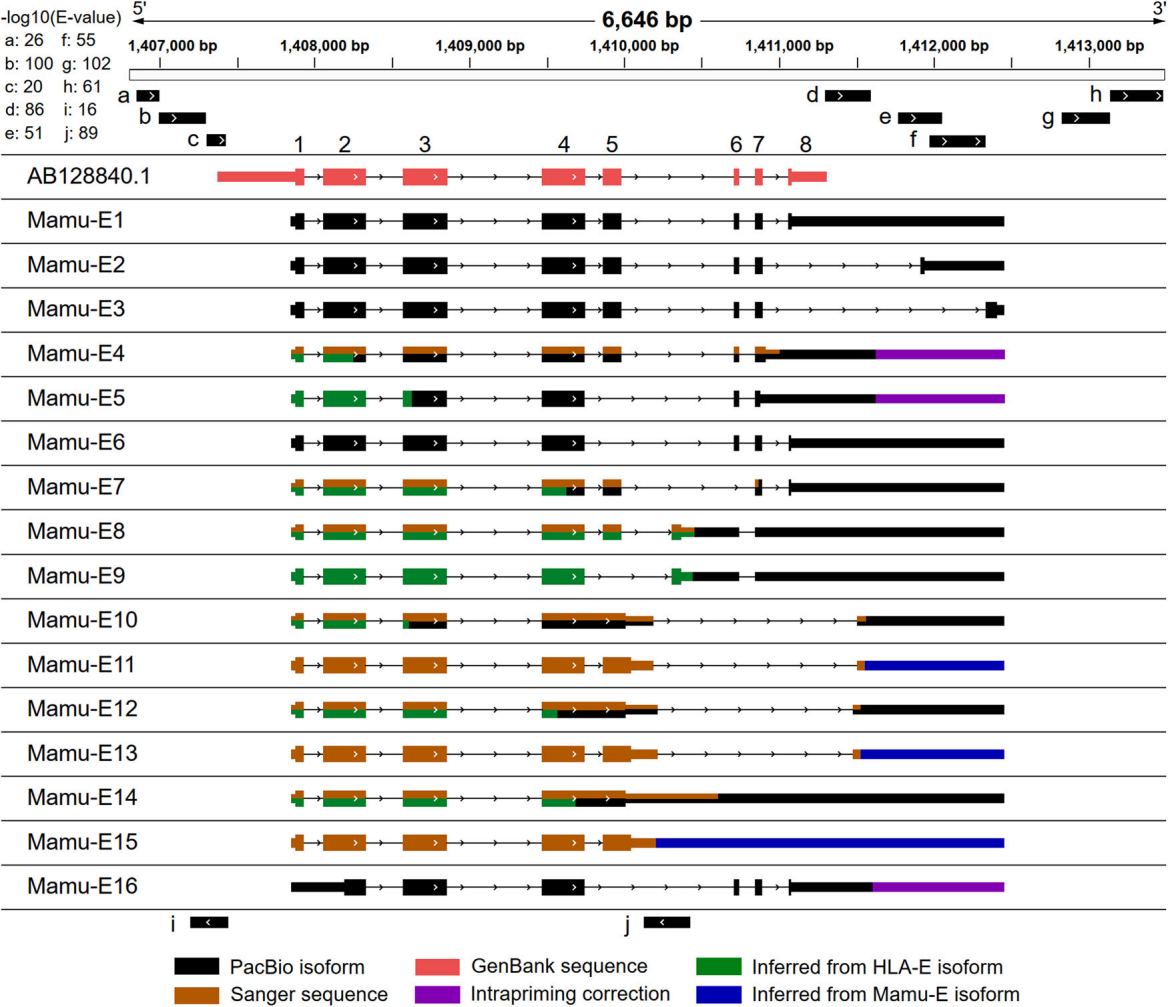
*Mamu-E* alternative splicing and retrotransposon activity. *Mamu-E* isoforms generated by Iso-Seq (black) and AB128840.1 (coral, previously reported *Mamu-E* cDNA) are shown by their genomic alignments. Additional Sanger sequence support (brown), inference from *HLA-E* isoforms (green), and extension of shortened 3′ end caused by intrapriming (purple) are also shown. Additional isoforms detected by Sanger sequencing (*Mamu-E11*, *13*, and *15*) are shown with 3′ ends inferred (blue) from matched *Mamu-E* isoforms. In cases where isoforms have support and/or inference from two sources, both colors are shown. Exons are shown as rectangles with lines representing introns removed by splicing. Thicker segments represent the coding regions of transcripts. Transcripts are shown from 5′ (left) to 3′ (right). AB128840.1 and *Mamu-E1* each represent the canonical splicing with exons 1–8 labeled above. Exons have the following correspondence with protein domains: 1 = signal peptide, 2 = Alpha 1 domain, 3 = Alpha 2 domain, 4 = Alpha 3 domain, 5 = transmembrane domain, 6/7 = cytoplasmic domain. Exon 8 is predominantly the 3′ UTR. Retrotransposons near or within the *Mamu-E* locus are shown as black segments above (sense strand) and below (antisense strand) the isoforms and are labeled with letters: a = AluJb, b = AluY, c = FLAM_C, d = AluY, e = MSTC, f = MLT-int, g = AluYf1, h = MSTD, I = SVA_F, j = AluSx3. E-values reported by the Dfam database are shown in the upper left.

To accurately define *Mamu-E* transcript structures, we aimed to use high-quality, full-length transcript sequences obtained by long-read transcriptome sequencing^41^. Since the sequences of MHC genes are very similar, it was critical that we use long-read sequencing to avoid transcript sequence assembly. In our previous work^42^, using PacBio transcriptome sequencing (the Iso-Seq method), we obtained over 2.8 million circular consensus sequencing (CCS) reads from four different rhesus macaque tissues (Supplementary Table 1). About 33% of these CCS reads were full length (i.e., contained the 5′ cDNA primer, 3′ cDNA primer, and polyadenylation tail), each representing a single transcript molecule^43^. All CCS reads (full-length (FL) and non-full length) were initially clustered without a genome reference and subsequently aligned to an RM MHC Class I region reference sequence, which was previously assembled using BAC cloning (Methods). These CCS read groups were further clustered and curated, yielding an initial set of 13 unique *Mamu-E* isoforms (shown in Fig. 1 as *Mamu-E1-10*, *12*, *14*, and *16*). The canonically spliced *Mamu-E* isoform (*Mamu-E1*) had the strongest FL CCS read support of all isoforms (92 of 123, 74.8%), while other isoforms had FL support ranging from 1 to 13 (Supplementary Table 2). Collectively, these isoforms supported a shorter 5′ UTR than previously annotated and a significantly longer 3′ UTR, and this was also supported by mRNA-seq data from RM whole blood samples described later (Supplementary Fig. 1). These isoforms exhibit several new splicing events largely concentrated at the 3′ end of the transcript, including exon skipping, alternative 3′ UTR splicing, a retained intron, and an unannotated exonization event between exons 5 and 6 (Fig. 1), all of which had canonical splice signals. Many of these isoforms were also predicted to encode protein sequences with different domain configurations (Fig. 1). For example, while nearly all isoforms (12 of 13) encode the canonical Alpha 1, 2, and 3 domains, many isoforms skip the transmembrane domain and have diverse cytoplasmic tails introduced by alternative 3′ UTR splicing. Together, these results indicate that complex alternative splicing of *Mamu-E* yields proteins with potentially diverse functions.

Separately from our *Mamu-E* analysis, we recovered 41 unique *HLA-E* isoforms collectively supported by 2050 FL CCS reads from a human PacBio Iso-Seq dataset from 60 myelogenous patient samples (Supplementary Fig. 2). Interestingly, all new splicing patterns in *Mamu-E* isoforms were found among these *HLA-E* isoforms, with 4 perfect isoform matches. Additionally, the human 3′ and 5′ UTRs were of comparable length to those in RM. A similar pattern was also observed among *HLA-E* isoforms, where most FL reads (1788 of 2050, 87%) supported the canonical splicing configuration. When resampling *HLA-E* isoforms using sequencing depth commensurate with *Mamu-E* (i.e., 123 FL CCS reads), 12.6 isoforms were detected on average, suggesting *HLA-E* and *Mamu-E* may have similar spliceosome complexities. Despite the greater number of isoforms detected in humans (41 vs. 13), only 25 contained the Alpha 1 and 2 domains needed for peptide binding (Supplementary Fig. 2, Supplementary Table 3). There were also many retained intron events detected between exons 1 and 2 (19 of 41 isoforms) of *HLA-E* compared to *Mamu-E* (1 of 13). Since here we sequenced samples from cancer patients, and intron retention is common among cancer samples^44^, some of these retained introns may be a characteristic of cancers. While the retained intron led to a frameshift and a premature stop codon in the *Mamu-E* isoform, this did not affect the reading frame in human isoforms (Supplementary Fig. 2). Further, while 3′ UTR splicing diversity was evident in humans, it did not impact the cytoplasmic tail, as the *HLA-E* open reading frame (ORF) terminates before the last splice junction (i.e., in exon 7); whereas *Mamu-E* isoforms terminate shortly after the junction (i.e., in exon 8) due to different exon 7 reading frames (Fig. 1, Supplementary Fig. 2).

Several *Mamu-E* isoforms with few FL CCS read support (8 of 13) captured new splicing patterns but failed to recover the complete *Mamu-E* 5′ end to varying degrees (Fig. 1). Given the consistency of the *HLA-E* and *Mamu-E* exon structures, we inferred 5′ ends for these incomplete isoforms. Next, we designed PCR assays to target the unique splicing features of these inferred isoforms and isolated the resulting bands for Sanger (Supplementary Table 4, Methods). The 5′ ends of most isoforms (6 of 8) were confirmed using this approach, and unexpectedly we identified three new isoforms (Fig. 1; *Mamu-E11*, *13*, and *15*). These isoforms match PacBio-derived isoforms (*Mamu-E10*, *12*, and *14*, respectively), but lack a retained intron between exons 4 and 5 (Fig. 1).

We hypothesized that this complex alternative splicing might be, in part, associated with transposable elements (TEs) in the *Mamu-E* locus. TE sequences are known to permeate the MHC region in RM^45^ and human^46,47^, and they are believed to play a significant role in human disease^48,49^. Alu elements, a type of transposon, have a strong connection with transcriptional regulation, as they can influence alternative splicing^50,51^ and function as enhancers^52^. We screened the *Mamu-E* locus and upstream and downstream genomic regions, finding eight elements on the sense strand and two on the antisense strand (Fig. 1). Interestingly, all eight of these TEs were found in the *HLA-E* locus in similar locations in the Dfam release 3.1^53^, suggesting these were translocated prior to the split between old and new world monkeys. Two Alu elements (AluJb and AluY) were found directly upstream of the 5′ UTR **(**Fig. 1), suggesting a possible role in transcriptional activation. We also detected an Alu element (AluSx3) on the antisense strand between exons 5 and 6, coincidentally where six isoforms (*Mamu-E8–13*) have unannotated splicing acceptor/donor sites that result in exons partially spanning the Alu element. Another AluY and two other TEs were found in the 3′ UTR, suggesting that alternative splicing in this region might be influenced by and/or influence their function. Lastly, mammalian-wide interspersed repeat (MIR)b was found directly downstream of the transcriptional termination site with a fully intact AluYf1 element directly adjacent to it (Fig. 1). Like Alu elements, MIRs can function as enhancers to promote tissue-specific gene expression^54^, and there is also evidence that they can be transcribed in human^55^. Taken together, the presence of complex splicing and deluge of TEs indicate that the *Mamu-E* and *HLA-E* loci are under strong transcriptional regulation.

### *Mamu-E* gene duplications are common

**Fig. 2:**
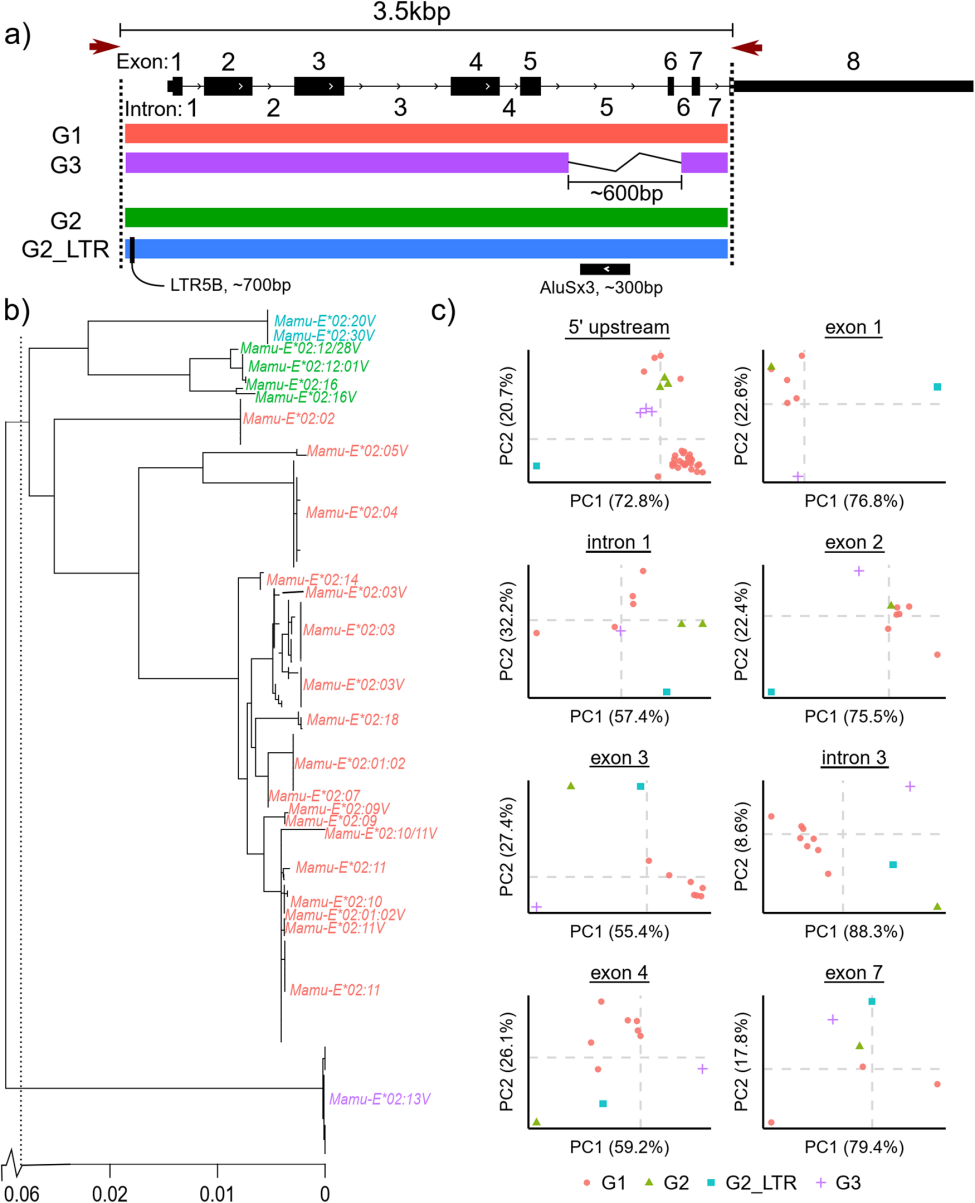
Genetic divergence of *Mamu-E* alleles. **a** Schematic showing the 3.5 kbp genomic region covered by PacBio sequencing of *Mamu-E* alleles. The exon–intron *Mamu-E* architecture is shown to scale with the four allele groups detected shown below in different colors: G1 (red), G3 (purple), G2 (green), and G2_LTR (blue). G2 and G2_LTR alleles are ~5% divergent from G1 alleles, and the latter harbors a ~700 bp LTR5B directly upstream of exon one. G3 alleles differ by a 600 bp deletion encompassing most of intron five and all of exon six. **b** Maximum likelihood tree of *Mamu-E* alleles, where branch lengths are scaled based on the expected number of mutations per nucleotide in the sequence. A break in the scale is used to concisely show the branches to distant alleles. Alleles are colored by their group designations described in (**a**) and are named based on the closest match(es) in the IPC-MHC database. A single label is used for identically assigned alleles adjacent to the tree. **c** Multi-dimensional scaling using pairwise sequence identities of selected allele segments highly divergent among allele groups. Each dot represents an allele. Data points were jittered to enhance visibility only in cases where those from different allele groups were completely overlapping (i.e., had identical coordinates). Data points from the same allele group may be overlapping.

*Mamu-E* has long been known to be polymorphic^17^, currently with 33 alleles in the immuno polymorphism MHC Database (IPD-MHC)^56^. To date, it has not been investigated whether this polymorphism has any connection with Mamu-E-restricted antigen presentation in response to RhCMV/SIV vaccination. We obtained genomic DNAs from 59 of 60 animals from four RhCMV/SIV vaccine groups, three previously described by Barrenäs et al.^30^, and used PacBio long amplicon analysis (LAA) to target and sequence *Mamu-E* allele sequences (Methods). Across 59 animals, we recovered 152 allele sequences (Supplementary Table 5), assigned to 17 IPD-MHC database alleles. These alleles were composed of four groups: three full-length ~5% divergent groups (G1, G2, G2_LTR) and a fourth monomorphic group missing the canonical *Mamu-E* exon 6 and the surrounding intronic sequence harboring an antisense AluSx3 element (G3) (Figs. 1, 2a, b). G2_LTR alleles are accordingly named by the ~700 bp solo LTR5B inserted approximately 20 bp after the expected start of the amplified sequence 5′ end (e-value ~ 10^−83^) (Fig. 2a). G1 alleles were detected in all animals and exclusively in 27 of 59 (46%), while additional alleles from G2, G2_LTR, and G3 were found in 6, 7, and 20 animals, respectively (Table 1).

**Table 1:** Allele groups detected in RhCMV/SIV study animals, stratified by protection outcome and group.

Protection outcome	Group	G1	G1 + G3	G1 + G2	G1 + G2_LTR	G1 + G2 + G2_LTR
Protected	O	4	4	1	0	0
	S	2	4	1	0	0
	X	4	1	1	0	0
Not protected	O	2	1	0	2	1
	S	3	2	0	2	0
	X	3	4	1	1	0
	E	6	4	1	1	0
Exposed-uninfected	E	3	0	0	0	0
Total		27	20	5	6	1

G2, G2_LTR, and G3 alleles were also found to be in complete linkage with Mamu-E*02:02, Mamu-E*02:11, and Mamu-E*02:04 (G1 alleles), respectively. We confirmed the presence of multiple *Mamu-E* loci in 1 of 4 selected animals (animal ID #Rh28808) using fosmid isolation followed by PacBio DNA sequencing (Methods, Supplementary Table 6). The fosmid sequence from this animal contained both the G3 allele (E*02:13V-short) and the E*02:04 allele separated by ~20 kb, supporting the linkage we observed between these alleles across multiple animals. No animals were found to have alleles from all four groups and or have >2 alleles from any of the groups, with the exception of one animal (animal ID # Rh29659). We recovered a third G1 allele (Mamu-E*02:03, also found in 11 other animals), which was not detected in our later expression analysis. The presence of additional *MHC-E* alleles in the same animals was not associated with vaccine group (Fisher′s exact test: p = 0.569) or protection outcome (Fisher′s exact test: p = 1) (Methods, Table 1), where the E group was excluded as there was no protection observed among its animals. However, all 7 animals from groups O, S, and X with G2_LTR alleles were not protected, and the association with protection outcome was statistically significant (Fisher′s exact test: p = 0.02, Table 1).

Next, we investigated the segments driving the sequence differences among allele groups by separately analyzing the exons, introns, and the sequence recovered upstream of 5′ UTRs. We observed that G2_LTR alleles significantly diverged from all other alleles even when removing the inserted LTR5b sequence (Fig. 2b, c). G1 alleles tended to cluster together in the 5′ upstream region, while a small subset clustered with G2 alleles and G3 alleles shared some similarities with both clusters (Fig. 2c). We found that G1 and G2 alleles were more similar in exons 1 and 2, while both G2_LTR and G3 alleles significantly diverged (Fig. 2c). Interestingly, all 4 allele groups diverged in exon 3 (Alpha 2), intron 3, and exon 4 (Alpha 3) (Fig. 2c), suggesting these allele groups may function differently.

### *Mamu-E* expression in whole blood is dominated by a single locus

To explore the potential functional divergences among duplicated *Mamu-E* alleles, we sought to determine if *Mamu-E* genes of these allele groups are similarly expressed. We examined *Mamu-E* gene expression using mRNA-seq analysis of whole blood samples collected from the same animals during the pre-challenge phase of an RhCMV/SIV vaccine study before and after the prime and boost phases (Methods). Nine samples from each of the 59 animals (531 total) were sequenced, yielding ~14.8 billion reads (~27.8 million reads per sample). For each animal, reads were aligned to the MHC Class I/II BAC reference with the *Mamu-E* locus masked and animal-specific *Mamu-E* allele sequences as separate contigs (Methods).

**Fig. 3:**
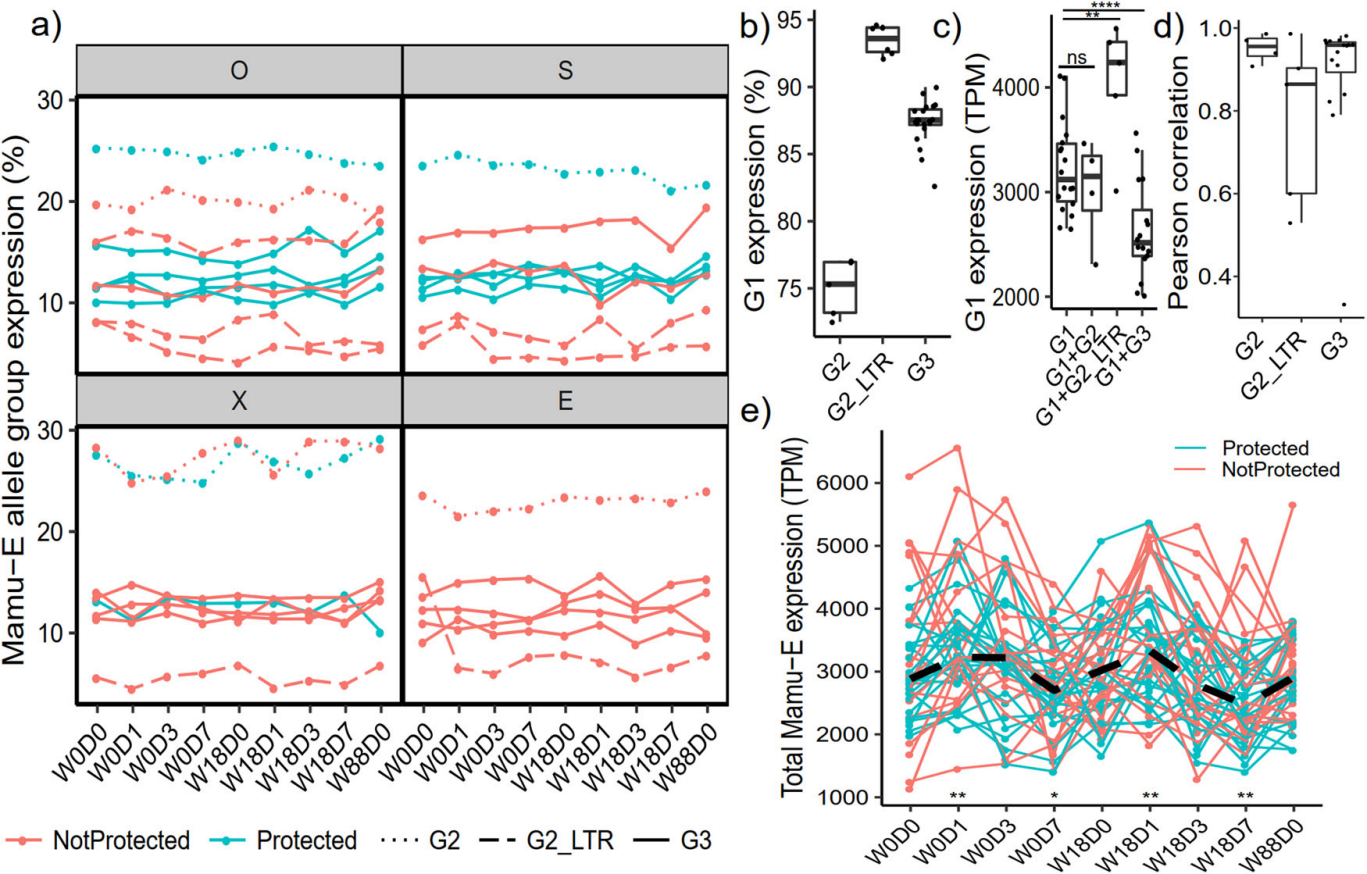
Proportion of *Mamu-E* allele group expression. **a** The proportion of *Mamu-E* expression from G2 (dotted line, *n* = 6), G2 with promoter LTR (G2_LTR, dashed line, *n* = 6), and G3 (solid line, *n* = 20) alleles is shown over the course of the pre-challenge phase for animals expressing alleles from these groups. Alleles from animals later protected from SIV infection are shown as blue lines, while those not protected are shown as red lines. Animals are stratified into quadrants based on their vaccine groups (O, S, X, and E). **b**, **c** Average *Mamu-E* expression from G1 alleles stratified by the allele groups present in animals (G1: *n* = 18, G2: *n* = 5, G2_LTR: *n* = 6, G3: *n* = 20). The proportion of G1 allele expression is shown in (**b**), while expression is shown in transcripts per million (TPM) in (**c**). **d** For each animal with an allele from the second group of alleles (G2, G2_LTR, or G3), the spearman correlation is computed between G1 and the second group using all pre-challenge time points. **e** Total *Mamu-E* expression, where each line represents an animal that is protected (blue, *n* = 22) or not protected (red, *n* = 21). The black dashed line indicates the median expression trend. Significance is determined by comparing expression at timepoint with baseline (W0D0). Since no difference was observed between protected and non-protected animals, significance tests were performed with all animals together. In **b**–**d**, boxplots are defined by quartiles (upper, median, lower) with whiskers extending to data points up to 1.5 times the interquartile range. In all cases **b**–**c**, **e** Wilcoxon rank-sum tests were used. ****p < 0.001, ***p < 0.01, **p < 0.05, *p < 0.1, ns = not significant.

We calculated the relative expression of allele groups in all animals expressing at least 1 allele from more than one group based on our genomic analysis (Table 1). The proportions of expression from each allele group were fairly stable throughout the pre-challenge phase, with G2, G2_LTR, and G3 alleles composing approximately 25%, 10-15%, and 5% of expression, respectively (Fig. 3a). While G1 alleles composed most of the *Mamu-E* expression, both the relative (Fig. 3b) and absolute (Fig. 3c) G1 allele expression levels varied contingent on the extra allele groups present in the same animals. For example, when G2_LTR alleles were present, the absolute G1 allele expression levels were about 30% higher (Fig. 3c). However, when G3 alleles were present, the absolute G1 allele expression levels were about 30% lower.

RhCMV/SIV vaccination elicits MHC-E-restricted T-cell responses, so we next sought to determine the effect of vaccination on the expression of these alleles. We observed that in animals expressing alleles from >1 group, the expression of allele groups was strongly correlated (Fig. 3d). When examining total *Mamu-E* expression (i.e., pooled allele expression), we found that *Mamu-E* expression increased significantly following vaccination prime and boost, regardless of protection outcome (Fig. 3e), suggesting that RhCMV/SIV vaccination may influence the functions of *Mamu-E*.

We also examined the relative expression of alleles expressed within the same group, finding that we could reliably recover allele-specific read counts even with little polymorphism between alleles (Supplementary Fig. 3a). We also observed fairly even allelic coverage within loci regardless of allele group that was also stable throughout the pre-challenge phase (Supplementary Fig. 3b-c). One exception to this was in one animal (animal ID # Rh28835 from the S group), where one G1 allele was found to be expressed substantially less than the other (Supplementary Fig. 3b). Interestingly, the lowly expressed G1 allele was the only allele among all animals with an insertion, which incidentally resulted in a frameshift and premature stop codon. These results indicate that G1 alleles tend to be expressed at relatively similar levels to each other and several times higher than G2 and G3 alleles.

### Confirmation and extension of *Mamu-E* G1 alleles using mRNA-seq-based haplotype phasing

We independently assessed the accuracy of our *Mamu-E* allele sequencing at per base level and captured an additional 3′ UTR variation using the collected whole blood mRNA-seq data. Also, as shown in Figs. 1, 2a, *Mamu-E* transcribes a much longer 3′ UTR than the canonical annotation. This long 3′ UTR was not covered in our allele genomic sequencing designed to target coding regions (Fig. 2a). We focused this mRNA-seq-based analysis on alleles in the G1 group since their expression was dominant, making this effort feasible (Fig. 3a, b).

We first assessed the depth of mRNA-seq read coverage of *Mamu-E* and the ability to capture *Mamu-E* polymorphism accurately using short-read mRNA-seq data. We observed ~4-5% of total reads mapped to the Mamu Class I and II complexes and 10,000 per base *Mamu-E* coverage (Supplementary Fig. 1). We also found that recovery of the transmembrane domain region polymorphisms was intractable likely due to greater conservation of this region with other MHC genes using a kmer-based strategy (Supplementary Fig. 4a, Methods). Recovery of polymorphisms in 3′ UTR regions harboring TEs was also found to be intractable, leading to their exclusion (Supplementary Fig. 4b). Lastly, low coverage bases proximal to the transcriptional start and termination sites were excluded from this haplotype phasing analysis (Supplementary Fig. 4c).

For the remaining highly confident regions, we generated completely contiguous haplotype blocks spanning the entire *Mamu-E* region, resulting in *Mamu-E* haplotigs (Methods). Almost all (1,146 of 1,150, 99.7%) heterozygous variant calls were successfully phased for all animals. As expected, we did not observe a lower fraction of reading support for the G1 haplotype configuration in animals with additional G2 and G3 alleles (Supplementary Fig. 5a), given the dominant expression of G1 alleles. On average, almost 100% of the variants identified by haplotigs derived from mRNA-seq reads were identical to the most similar G1 allele sequences within each animal where they overlap (i.e., excluding the 3′ UTR) (Supplementary Fig. 5b). For each animal, we matched haplotigs against allele sequences, determining that variant phasing was also highly concordant (>96% of variants) with differences only arising due to mRNA-seq variant calling issues in fringe locations just passing our required per base coverage threshold (Supplementary Fig. 5c). This nearly perfect agreement between these two independent methods (DNA sequencing via PacBio LAA, haplotig recovery via mRNA-seq) shows the extremely high accuracy of sequences we obtained by LAA. We merged these G1 alleles with their matched haplotigs, producing final, complete G1 allele sequences spanning the entire *Mamu-E* locus, including both coding regions and long 3′ UTRs.

### Characteristics of *Mamu-E* G1 allele variants and their associations with RhCMV vaccine protection

**Fig. 4:**
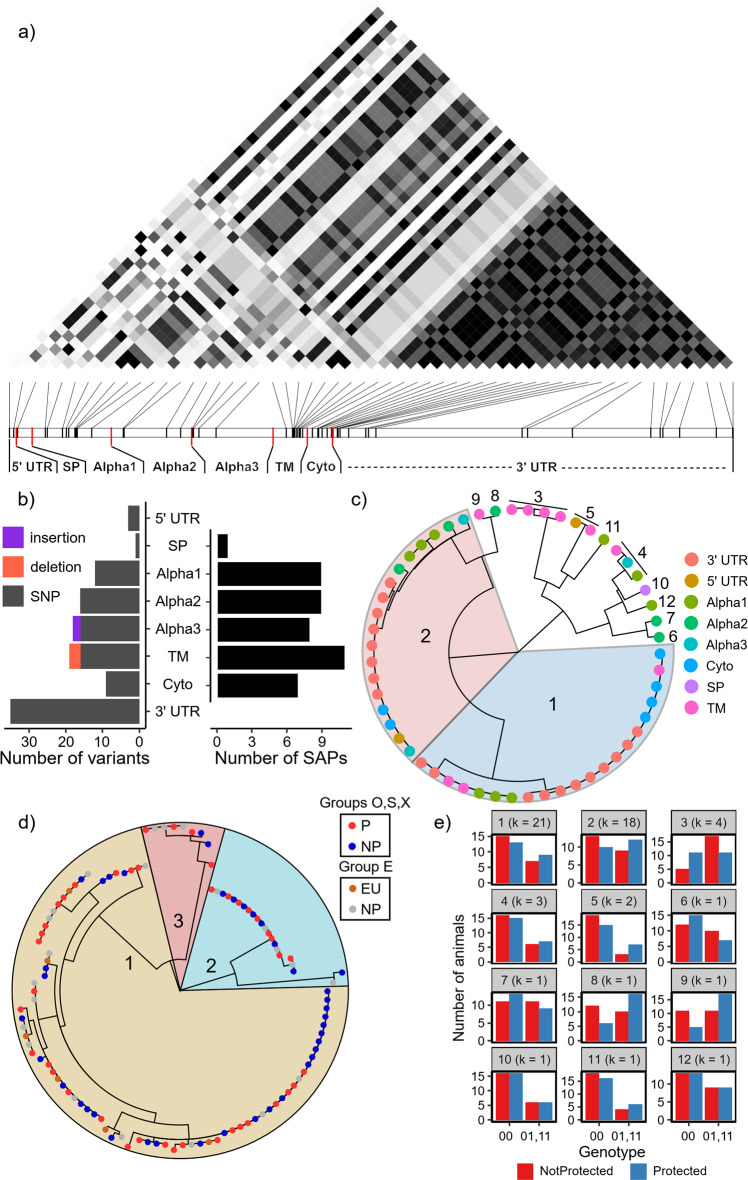
Genetic analysis of G1 alleles. **a** Heatmap showing linkage disequilibrium (LD) of variants, ordered by positions in the canonical *Mamu-E* transcript from left (5′) to the right (3′). Each entry indicates the LD between two variants, whereas darker entries are those with higher LD values. **b** A number of variants (left) and single amino-acid polymorphisms (SAPs, i.e., non-synonymous SNPs) in *Mamu-E* transcript regions. Variants are colored by type (gray = single nucleotide polymorphism (SNP), coral = deletion, purple = insertion). **c** Hierarchical clustering of variants based on their correlations, with correlated groups of SNPs numbered. Two large variant clusters are shown in blue (1) and red (2). **d** Maximum likelihood tree of the final G1 allele sequences, including 3′ UTRs from haplotigs. Major G1 allele subgroups are colored based on their overall grouping. Tips are colored based on the protection outcome of the animal from which the allele was recovered. **e** Number of protected (blue) and not protected (red) animals from vaccine groups O, S, and X carrying homozygous dominant (00) versus heterozygous (01) and homozygous recessive (11) variant clusters. Groups are ordered and labeled by their number of variants (*k*, i.e., SNPs, indels) as shown in panel (**c**), beginning with the largest in the top left.

Next, we examined the variation recovered across these merged G1 allele sequences, since all animals have at least one copy of G1 alleles and G1 alleles contributed the majority of *Mamu-E* expression in whole blood samples (Fig. 3a, b). Variants were identified throughout the whole *Mamu-E* G1 locus, protein-coding regions, and both UTRs (Fig. 4a, b). Single nucleotide polymorphisms (SNPs) were also found to be non-synonymous, producing a total of 42 unique single amino-acid polymorphisms (SAPs) spread across all protein domains (Fig. 4b). However, none of the SAPs located in the Alpha 1 and 2 domains were located in the predicted B and F pocket key binding sites^57,58^ (Supplementary Fig. 6). Interestingly, G2 and G3 allele polymorphisms also did not affect key binding sites. However, those in G2_LTR alleles impacted 5 sites across Alpha 1 and 2, indicating that they likely have significantly altered function.

Given the extent of polymorphism recovered among G1 alleles (108 SNPs, 2 insertions, 3 deletions), we decided to inspect individual variants and determine the extent of linkage disequilibrium (LD) within the *Mamu-E* G1 locus. We found that 55 of 113 (48.7%) passed a minor allele frequency (MAF) threshold of 0.1 (Supplementary Table 7). Variants passing the MAF filter were located throughout the 3′ UTR and coding region of the *Mamu-E* locus (Fig. 4a). We detected substantial correlation (i.e., LD) of variants both locally and between variants distant from one another in the *Mamu-E* G1 locus (Fig. 4a). When we grouped these variants based on their correlations, we found 2 major clusters of correlated variants of size 21 and 18 SNPs, with 10 additional clusters of size 4 or less (Fig. 4c). The only indel that passed the MAF filter (a deletion in the transmembrane (TM) domain) was in strong LD with 3 SNPs also in the TM domain (cluster 3 in Fig. 4c). Interestingly, the two large clusters of SNPs were each comprised of a set of 3′ UTR variants along with variants from the Alpha domains, TM domain, cytoplasmic domain, and 5′ UTR (Fig. 4c). We also observed that when represented in a phylogeny, final G1 allele sequences formed 3 major subgroups, with one much larger than the other two (Fig. 4d). However, there was no significant association between these G1 allele subgroups and vaccine group (Fisher′s exact test, p = 0.303) or protection outcome (Fisher′s exact test: p = 0.313). We then cross-referenced the variant clusters identified (Fig. 4c) with the three major G1 allele subgroups identified (Fig. 4d), finding that G1 subgroup 1 alleles contained the major form of both of the two large variant clusters, subgroup 2 contained the minor form of both, and subgroup 3 contained the minor and major form of the first and second, respectively.

We then examined the genotypes of animals across each of the variant clusters as well as individual variants, finding that there was no statistically significant association with protection outcome among vaccine groups O, S, and X (Fig. 4e, Supplementary Table 7) or with vaccine groups (Supplementary Fig. 7, Supplementary Table 7). However, we did observe a tendency for protected animals to favor the major form of variant cluster 3 and the minor forms of variant clusters 8 and 9 (p = 0.116, 0.124, 0.116 and false discovery rate (FDR) = 0.497, 0.497, 0.497, respectively) (Fig. 4e, Supplementary Table 7).

**Fig. 5:**
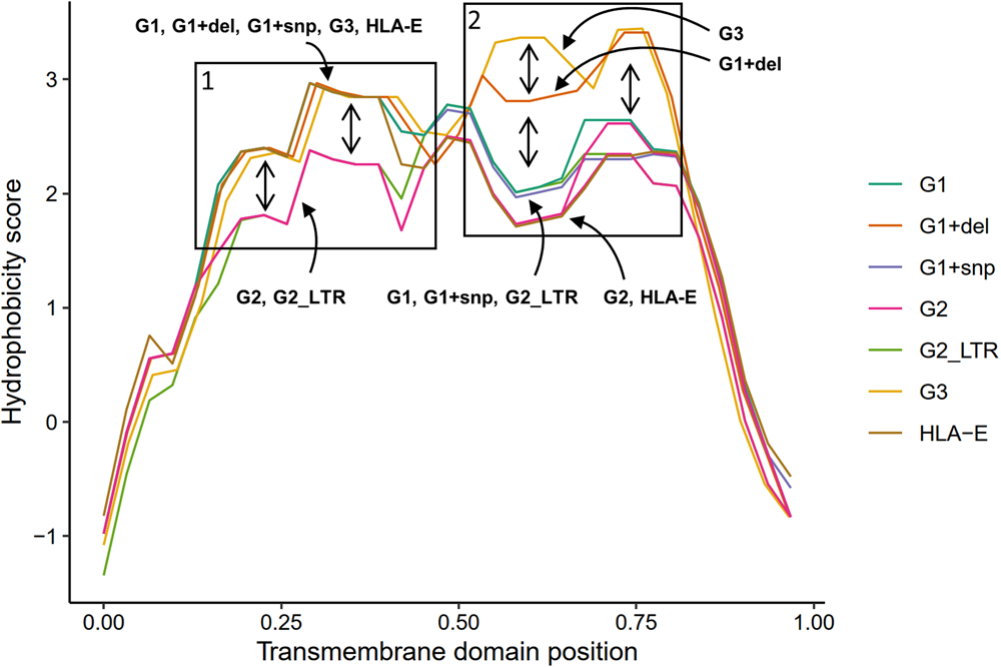
Predicted hydrophobicity scores for *Mamu-E* Transmembrane (TM) domains. Positions are normalized in the 0–1 range to account for differences in TM domain length due to indel variation. G1 alleles are shown in turquoise, while those harboring a deletion from variant cluster 3 and those with an SNP (cluster 9) are shown in orange and purple, respectively. G2, G2_LTR, and G3 alleles are shown in pink, green, and yellow, respectively. These are compared to the only known *HLA-E* TM domain shown in brown. G2 and G2_LTR TM domains have lower N-terminal predicted hydrophobicity (i.e., a normalized position less than 0.5) relative to all other alleles, denoted by arrows and box 1. G3 and G1 alleles with a deletion have higher C-terminal predicted hydrophobicity, denoted by arrows and box 2. G3 alleles have even higher hydrophobicity in part of this denoted region. All hydrophobicity scores were computed using Expasy ProtScale with the Kyle & Doolittle scale.

Since cluster 3 harbored 4 variants (including a deletion) in the TM domain, as did variant cluster 9 (SNP), we examined the differences in hydrophobicity scores across all allele TM domains. We observed a reduction in N-terminal TM hydrophobicity among all G2_LTR and G2 alleles relative to G1 alleles and *HLA-E* (Fig. 5). We also found that G3 alleles and G1 alleles harboring variant cluster 3 and had increased C-terminal TM hydrophobicity relative to other *Mamu-E* alleles as well as *HLA-E*, while *Mamu-E* alleles with cluster 9 had unaltered hydrophobicity (Fig. 5). These results suggest that *Mamu-E* polymorphisms in this region may impact Mamu-E protein transport and/or membrane stability. Furthermore, we cannot make a complete determination since variants located within 3′UTR TEs and indels in the 3′ UTR region were not included in this analysis (Supplementary Fig. 4b).

Since we determined that the *Mamu-E* expression in whole blood was driven by G1 alleles, we investigated if the G1 allele expression, especially isoform usage, in whole blood could be related to RhCMV/SIV vaccine protection outcome. The abundances of *Mamu-E* G1 allele isoforms were quantified using full G1 allele sequences, and relative proportions of isoforms were determined for all pre-challenge time points for each animal (Methods). We found that *Mamu-E1* isoforms were most prevalent, composing ~80% of isoform abundances, while all other isoforms were detected at lower levels (Supplementary Figs. 8, 9). We also observed that relative isoform usages were largely stable in whole blood throughout the pre-challenge phase, regardless of vaccine protection outcome and vaccine group (Supplementary Fig. 9). *Mamu-E* isoforms were observed in different strata based on their relative expression (Supplementary Fig. 8). *Mamu-E2*, *4*, *6*, *8*, and *14-16* formed a second stratum after *Mamu-E1*, each representing ~1-10% of isoforms expressed. *Mamu-E3*, *5*, *7*, *9*, *10*, and *12* formed the third stratum, each expressing ~0.1-1% of isoforms. *Mamu-E11* and *13* were especially rare, defined as the fourth stratum with <0.1% of isoform expression. Incidentally, these two rare isoforms were only identified using Sanger sequencing (Fig. 1).

## Discussion

Here we present the first comprehensive analysis of alternative splicing and genetic variations across the whole *Mamu-E* locus. We used long-read sequencing of both RNAs and DNAs to address the genomic complexity in the rhesus *MHC-E* region and complementary mRNA-seq analysis for independent validation and expression quantification. We uncover complex *Mamu-E* alternative splicing that is also conserved in humans. We show that the whole *Mamu-E* locus is polymorphic, and *Mamu-E* gene duplications are common, a striking contrast compared to the highly monomorphic *HLA-E* in humans.

Up to this point, the standard annotation of both *HLA-E* and *Mamu-E* has been a single transcript with the canonical MHC Class I exon/intron splicing, but the evidence we describe suggests that *MHC-E* transcription is regulated by complex alternative splicing. Interestingly, all *Mamu-E* splicing junctions were also found in *HLA-E* splicing isoforms. The high conservation of alternative splicing between *Mamu-E* and *HLA-E* provides additional evidence that rhesus may serve as a good model for studying *HLA-E* immunobiology. However, it also suggests that further investigation of these isoforms is needed to better understand the regulation and function of *MHC-E* in both RMs and humans.

Historically, MHC genotyping analyses have focused on coding regions, yet the substantial polymorphism in the *Mamu-E* 3′ UTR, the presence of TEs, and the 3′ UTR alternative splicing we observed in this study warrant further investigation and more expanded genotyping efforts. In the future, we anticipate expanding the LAA approach employed here to cover the full range of the 3′ UTR annotated in this study. Moreover, tapping into TEs in regions surrounding MHC genes might also inform research on MHC gene duplications in RM, as we observed with *Mamu-E* G2_LTR alleles in this study.

Since our original PacBio LAA design did not cover the *Mamu-E* 3′ UTR, we explored the feasibility of recovering genotype information in that region using available mRNA-seq data for those animals. MHC genes, *Mamu-E* in particular, are constitutively expressed, making RNA-seq coverage less problematic than for typical protein-coding genes. There is also a strong propensity for splicing observed here with *Mamu-E* and *HLA-E*, but also with many other MHC genes^59^. Splicing uniquely provides long-range haplotype information, a clear advantage over high-throughput DNA-seq. We were able to phase nearly all heterozygous variants detected despite blacklisting portions of the 3′ UTR containing repetitive TE-derived sequence. This type of in silico approach is common, originally explored for HLA typing with the seq2HLA tool^60^ and, more recently, with the arcasHLA tool^61^. We expect this strategy to be useful for the re-analysis of previously published RM mRNA-seq data for examining the prevalence of known and potentially novel *Mamu-E* allele sequences among different RM colonies.

We present direct evidence that *Mamu-E* gene duplications are common, detecting them in ~50% of 59 animals we sequenced here. Since there is no reported *HLA-E* gene duplication and *HLA-E* is highly monomorphic in humans, our discovery of widespread *Mamu-E* gene duplications and polymorphisms raises questions about the translational potential of the rhesus model in the context of *MHC-E*. We suspect the functions of *MHC-E* are mostly conserved between RMs and humans, but there may be large variations among RMs depending on the *MHC-E* genetics of individual animals. For example, overall, we did not find noticeable associations of genetic variations with RhCMV/SIV vaccine protection, though the total number of animals was relatively small. We did observe the presence of G2_LTR alleles was significantly associated with the lack of RhCMV/SIV vaccine protection, but this subset of animals was relatively small (6 out of 59, ~10%). Wu et al. suggested potential duplications of the *Mamu-E* locus based on the expression of multiple *MHC-E* transcripts within individual RMs, but they did not observe significant functional differences among *Mamu-E* molecules^20^. We detected dominantly expressed G1 alleles in whole blood samples, and G1 alleles were present in all animals. Further, in whole blood samples, we found that the canonical *Mamu-E1* isoform was most abundant, and all other isoforms collectively composed ~20% of expression. This suggests that the dominant *Mamu-E1* isoform from the common G1 alleles may drive the general functional conservations between rhesus and humans. However, our results show that *Mamu-E* expressions also appear to be dose-sensitive, suggesting potential interactions among *Mamu-E* alleles.

Clearly, more work will need to be done to examine *Mamu-E* allele and isoform expression patterns in other tissues and cell types with different phenotypes. For example, *Mamu-E* alternative splicing could be investigated at the single cell level potentially with 3′ tag approaches using the 10x Genomics platform, as much of the splicing diversity is concentrated at the 3′ end. This 3′ alternative splicing was found to affect the inclusion of the 3′ UTR and also impacted the protein sequence of the cytoplasmic tail, but only in Mamu-E, as the HLA-E protein sequence terminates before the exon 7-8 junction. Cytoplasmic tails are believed to be important for selective export from the endoplasmic reticulum, and there is supporting evidence in the case of *HLA-F*^62^. Manipulation of cytoplasmic tails of another MHC Class I molecule, *Patr-AL*, drastically affected its surface expression^63^. It was also shown that splice variants of MHC class I molecules resulting in the deletion of amino acids in exon 7 improved the CD8+ T-cell stimulatory capacity of DC cells^64^. Collectively, this body of evidence suggests that such *Mamu-E* splice variants affecting the cytoplasmic tail could generate proteins with different functional outcomes.

This study is the first to interrogate both the genetics and alternative splicing of *Mamu-E* with this level of precision in the context of an RhCMV/SIV vaccine study. The surprising association of *Mamu-E* G2_LTR alleles with the lack of RhCMV/SIV vaccine protection and the weak associations between selected *Mamu-E* variants and RhCMV/SIV vaccine protection should be followed up. Our genetic analysis missed a few highly variable regions in the 3′ UTR due to limitations of mRNA-seq haplotype phasing. A closer examination of these specific variants and regions in the future will offer a better understanding of the potential impact of *Mamu-E* genetic variations. The analysis of isoform usages was complicated in that we used mRNA-seq analysis of whole blood samples, which included many cell types. Additional analysis of specific cell types or even single cells may be necessary to fully investigate if alternative splicing plays any role in RhCMV/SIV vaccine-induced protection. It is our belief that this study lays the groundwork needed for a more comprehensive analysis of *Mamu-E*, which in turn will facilitate a more informed assessment of RhCMV-based vaccine translatability as we look toward hCMV/HIV vaccine development.

## Methods

### Ethical statement

RM care and all experimental protocols and procedures were previously approved by the ONPRC Institutional Animal Care and Use Committee^30^. The ONPRC is a Category I facility. The Laboratory Animal Care and Use Program at the ONPRC are fully accredited by the American Association for Accreditation of Laboratory Animal Care and has an approved Assurance (#A3304-01) for the care and use of animals on file with the NIH Office for Protection from Research Risks. The ONPRC adheres to national guidelines established in the Animal Welfare Act (7 U.S.C. Sections 2131–2159) and the Guide for the Care and Use of Laboratory Animals (8th Edition) as mandated by the U.S. Public Health Service Policy.

Pediatric AML biological samples were previously collected with informed consent (and in accordance with the Declaration of Helsinki) from patients diagnosed with de novo AML and enrolled in Children’s Oncology Group (COG) trials AAML0531 (NCT00372593), or AAML1031 (NCT01371981)^65^. Each protocol was approved by the National Cancer Institute′s central institutional review board (IRB) and the local IRB at Fred Hutchinson Cancer Center (Protocol 9950).

### Rhesus full-length transcriptome sequencing and data processing

Full-length transcriptome sequencing data were generated from four rhesus tissues (whole blood, peripheral blood mononuclear cells, lymph node, and rectal biopsy) and pre-processed in our previous work to produce circular consensus sequence (CCS) reads^42^. CCS reads were then aligned to Mamu Class I and II assemblies previously generated using Bacterial Artificial Cloning (BAC) technology^66^ (AC148696.1) and annotated using *Mamu* and *HLA* cDNA and protein sequences available in GenBank. STARlong v2.5.2b^67^ was used for alignment with the following parameters specified: –alignEndsType EndToEnd, –outFilterMismatchNoverReadLmax 0.05, –outFilterMatchNminOverLread 0.95, –twopassMode Basic, –outFilterMultimapNmax 20, –outFilterIntronMotifs RemoveNoncanonical, –outFilterType BySJout. To mitigate the splicing of reads between highly distant yet similar *MHC* loci, we serially aligned CCS reads, gradually increasing the maximum intron length using the –alignIntronMax parameter with the following values: 5000, 15000, 100000, 0 (no maximum). CCS reads that successfully aligned to the BAC reference were further processed using the Iso-Seq bioinformatics pipeline^41^ and its supporting Cupcake scripts (https://github.com/Magdoll/cDNA_Cupcake) to produce full-length (FL) consensus isoforms. FL *Mamu-E* isoforms were realigned to the BAC reference and then curated by correcting splice junctions misaligned due to indel events, extending 3′ ends shortened by intrapriming in the 3′ UTR, and collapsing any redundancies in the isoforms produced by these corrections. Finally, the transcriptional start and termination sites (TSS, TTS) for isoforms were clustered using a window size of 50 nucleotides. For isoforms within each cluster, the TSS or TTS was updated to match that of the isoform that extended the annotation to the furthest (smallest and largest genomic coordinate for TSS and TTS, respectively).

### Human full-length transcriptome sequencing and data processing

RNA was isolated from 60 samples of myelogenous cells obtained from human patients^65^. Using a Clontech SMARTer kit, cDNA was produced from each RNA sample, followed by PCR amplification. Libraries were prepared using the SMRTbell Express Template Prep Kit 2.0 and sequenced on the Sequel II System (Pacific Biosciences, Menlo Park, CA). Raw PacBio data were first pre-processed using the CCS protocol^68^ to generate a complete set of CCS reads. CCS reads were then aligned to the human genome (hg38) and subsequently processed using the Iso-Seq pipeline as described above, and the resulting isoforms were characterized using SQANTI^69^. *HLA-E* isoforms were then realigned to hg38 and curated as described above for *Mamu-E* isoforms. Additionally, short isoforms with both a TSS and TTS located within introns (classified as genic introns or genic genomic isoforms by SQANTI) were removed, as they were likely sequencing artifacts or fragmented mRNAs. To compare *HLA-E* and *Mamu-E* spliceosome complexities, *HLA-E* isoforms were sampled with replacement using their respective FL read counts to estimate the probability of detection. This was repeated 10,000 times using the total number of *Mamu-E* FL read counts each time, and the mean and standard deviation of the results were recorded.

### Cross-species comparison of *MHC-E* isoforms

To facilitate cross-species comparison of *MHC-E* isoform structures, the genomic DNA of the *HLA-E* and *MHC-E* were aligned to each other, and a genomic coordinate converter was generated from the alignment. *Mamu-E* isoform genomic coordinates were thus converted to *HLA-E* coordinates and compared to those of *HLA-E* isoforms. *Mamu-E* isoforms with incomplete 5′ end but otherwise complete matches to *HLA-E* isoforms had their 5′ ends inferred using the *HLA-E* 5′ ends.

### Validation of inferred *Mamu-E* 5′ ends using PCR and Sanger Sequencing

To validate inferred 5′ ends of select *Mamu-E* isoforms, isoform-specific PCR assays were designed. In brief, a common forward primer targeted the canonical first *Mamu-E* exon, while the reverse primers were isoform-specific (Supplementary Table 4). In cases where a reverse primer could not be designed uniquely for an isoform, the primer was designed to produce an amplicon of unique size for the isoform of interest. cDNA was obtained from whole blood RNA pooled from multiple RMs (Qiagen QuantiTect RT) and amplified using TD-PCR to help limit off-target effects (Agilent Herculase II Fusion Polymerase). Most commonly, phase 1 consisted of 10 cycles started at an annealing temperature (T_a_) of 65 celsius (C) that was reduced to 1 C per cycle. Phase 2 utilized a T_a_ of 56 C for an additional 30 cycles. The extension was performed at 25 s. PAGE-based gel purification was performed on selected amplicons which were eluted overnight in 100 µL 0.1× TAE at room temperature on an orbital shaker. Eluted bands were concentrated via centrivap and then re-amplified and purified to increase yield and purity. Each amplicon was examined via PCR using sequencing primers paired with the appropriate PCR primer to help eliminate any bands that were products of PCR bubbling and to reconfirm band sizing before sequencing. Purified bands were then Sanger sequenced at Eton Biosciences, Inc. using the same primers used for PCR. In cases where the band size exceeded Sanger Sequencing limitations, forward and reverse primers were designed in the canonical *Mamu-E* exon 4 to pair with the PCR primers and produce two overlapping sequences for the band. The resulting sequence trace files were imported into SnapGene and exported to produce fastq files. In cases where multiple sequences were produced for a single band, sequences were merged using PEAR v0.9.10^70^ with default parameters. Final merged sequences were then aligned to the expected amplicon sequence for the band. Unexpected, unannotated *Mamu-E* isoforms generated from these assays were added to the existing isoform annotations.

### Isoform functional analysis and identification of genomic TEs

*Mamu-E* and *HLA-E* isoforms were each analyzed for coding potential. Isoform cDNA sequences were extracted from the respective reference sequences using the isoform GTF annotation file and the gffread tool from cufflinks v2.2.1^71^. Consensus domain sequence (CDS) annotation was then generated by aligning these sequences back to the reference using GMAP v2019-21-01^72^ with the –format=gff3_gene, -z sense_force, and -F parameters. These CDSs were then extracted using gffread and translated into protein sequences.

Separately, the entire Mamu Class I and II BAC reference sequences were screened for TEs using Dfam release 3.1^53^ with the organism set to Homo Sapiens. Database hits were then parsed to produce GTF records that were visualized together with *Mamu-E* isoform annotations using the Integrative Genomics Viewer^73^. These database hits were also compared to those pre-calculated at the *HLA-E* locus in Dfam release 3.1.

### RhCMV/SIV vaccine study sample collection

Whole blood PAXgene samples were collected from 3 vaccine groups of 15 male RMs each (oral 68-1 vaccination group O, subQ 68-1 vaccination group S, and subQ 68-1 + 68-1.2 vaccination group X), as recently reported^28,30^. Whole blood samples were similarly collected from an additional vaccine group of 15 male RMs (subQ 68-1.2 group E) from this same study. PAXgene samples were collected prior to immunization and at days 1, 3, and 7 post-prime vaccination (W0D1, W0D3, W0D7) and post-boost (W18D0, W18D1, W18D3, W18D7). An additional sample was collected before the start of the first SIVmac239 challenge (W88D0).

### PacBio LAA and data processing

We obtained genomic DNAs from 58 of 60 animals from the four RhCMV/SIV vaccine groups described above and used PacBio LAA to target and sequence *Mamu-E* allele sequences. Two *Mamu-E* genomic reference sequences (NW_015057580 and NC_041757) were used for the design of long-range PCR primers (Supplementary Table 8). Three different primer sets were designed from the flanking regions of *Mamu-E* to avoid allelic drops due to unanticipated variation at the primer binding sites. Each set of primers generated ~3.2–3.5 kbp products. Two-stage long-range PCR was used for target generation (stage 1) and indexing of amplicons (stage 2). PCR products were combined in equimolar quantities, pooled into a single tube, and the pooled product was processed using the SMRTbell Express Template Prep Kit 2.0 (Pacific Biosciences, Menlo Park, CA). The sequencing library was sequenced in a single SMRT cell on the Sequel II System (Pacific Biosciences, Menlo Park, CA).

Raw data were analyzed first by demultiplexing with Lima, followed by running LAA to generate amplicon sequences, both components of PacBio′s open-source SMRT Analysis software suite (Pacific Biosciences, Menlo Park, CA). CCS reads were mapped back to amplicon sequences using in-house derived cluster/match analysis. Low-quality and recombinant sequences were filtered out to generate final amplicon sequences, and *Mamu-E* gene annotation from the derivative amplicon sequences was accomplished using Geneious Prime (San Diego, CA).

### Confirmation of *Mamu-E* duplications via fosmid isolation and PacBio sequencing

Four animals were targeted for fosmid isolation and sequencing (Supplementary Table 6) using modifications of the approach described in^74,75^. Sequencing was performed on the Sequel IIe System using the Sequel II Sequencing 2.0 Bundle according to the manufacturer’s protocol (Pacific Biosciences, Menlo Park, CA). CCS corrected reads of over 30 kbp were targeted for analysis, and consensus sequences were derived from overlapping ZMWs using the Celera Assembler Canu 2.0. Resulting in complete fosmid sequences that were then screened for *Mamu-E* allele sequences using Geneious Prime (San Diego, CA).

### Whole blood mRNA-seq and *Mamu-E* haplotype phasing

As previously described by Barrenäs et al.^30^, cDNA libraries were prepared and sequenced for all whole blood samples, and resulting sequencing data was demultiplexed using Illumina bcl2fastq. Raw reads for each sample were aligned to the MHC Class I and II BAC reference using STAR v2.7.7a^67^ with the following parameters set: –alignIntronMax 5000, –alignMatesGapMax 5000, –outFilterMultimapNmax 50. Reads uniquely mapped to *Mamu-E* (mapping score = 255) were extracted from the alignment output using samtools^76^ followed by the bedtools intersect tool^77^, where the *Mamu-E1* GTF annotation was used. Per base coverage of the *Mamu-E1* sequence was then computed using bam-readcount v0.8.0 (https://github.com/genome/bam-readcount) with parameter -b 20 to only count bases with quality score ≥20 from reads with perfect mapping scores. Coverage was pooled across all nine pre-challenge samples for each animal, and the ends of the *Mamu-E1* sequence with coverage below 10,000 reads per base were excluded from the analysis.

The uniqueness of the *Mamu-E1* sequence was assessed using two strategies. Firstly, kmer libraries (*k* = 76 bp) were generated for all *Mamu-E* alleles, and the kmers were aligned to the BAC reference using STAR, as described above. The rate of uniquely mapped, multimapped, and unmapped kmers was then assessed. Secondly, the rate of uniquely mapped reads was examined from the mRNA-seq samples aligned as described above. From the initial mapping results (from STAR), per base coverage was computed as above using the parameters -b 0 and -q 255 (uniquely mapped read quality score for STAR). This was performed a second time with -q 0 to capture the total per-base coverage from which the per-base multimapping rate was inferred. From these analyses, additional regions were identified and excluded from haplotype phasing.

For each *Mamu-E1* position remaining, bases were called using a threshold of 25% coverage for each animal. Positions with a single call were labeled as homozygous, and those with more than one as heterozygous, and a VCF file was then manually generated and indexed using SAMtools^76,78^. Next, using the mapping results from all nine samples for each animal, haplotype blocks were generated using phASER^79^, a haplotype phasing tool optimized for RNA-seq data, with the following parameters set:–paired_end 1–mapq 255–baseq 20. The statistical test for variant connections was disabled using the parameter –cc_threshold 0, as a small fraction of reads (<5%) were expected from additional *Mamu-E* loci with lower expression. In cases where multiple haplotype blocks were produced, additional phasing was performed inferentially by comparing the relative coverage of haplotypes from each block. For two blocks to be merged, a perfect consensus was required across all nine samples. Any remaining heterozygous positions not included in the largest haplotype block were assigned an ambiguous call using standard IUPAC ambiguity codes (e.g., A or C = M). Haplotypes were then screened for variants with low phasing support by assessing variant connections with at least 100 read support. Variants that, on average, had >20% connections with other variants conflicting with the haplotype configuration were removed from the haplotype block and labeled as ambiguous calls. Lastly, haplotypes were expanded to include homozygous positions, resulting in complete *Mamu-E1* haplotig sequences.

### Comparison and integration of *Mamu-E* alleles with mRNA-seq haplotigs

Exons 1–7 were extracted from haplotigs and aligned with all allele exonic sequences using PRANK^80^, an indel-aware progressive multiple sequence aligner. In cases where haplotigs in an animal only differed by 3′ UTR SNPs, these were collapsed into a single haplotig at this step. Then, using this alignment, each haplotig was compared to each allele from the same animal, excluding indel variation captured by the alleles and regions blacklisted in the haplotype phasing analysis. Haplotypes and G1 alleles were progressively matched, taking the pairing with the fewest mismatches and subsequently pairing the remaining allele and haplotig, if any. G1 alleles were then merged with the 3′ UTRs of matched haplotigs by using the intronic sequence between exons 7 and 8 extracted from the MHC Class I/II BAC reference using gffread^71^, yielding a single contiguous sequence. All SNPs and indels detected in the alleles and the 3′ UTR of haplotigs were gathered for each animal into a single VCF file for later genetic analysis. All of these variants were also enumerated, stratifying over the different protein coding regions and both UTRs. Non-synonymous SNPs were separately counted.

### *Mamu-E* phylogenetic and genetic analysis

All multiple sequence alignments of alleles, including those of exonic and promoter regions, were performed using Clustal Omega^81^, a progressive multiple sequence aligner. PRANK^80^ was not used here, as Clustal Omega performed better when including noncoding regions. Phylogenetic analysis was then performed in R using the phangorn package^82^ for all multiple sequence alignments. In brief, a neighbor-joining (NJ) tree was generated using the dist.ml function with the multiple sequence alignment as input, followed by the NJ function. A maximum likelihood (ML) tree was generated from the NJ tree and multiple sequence alignment using the pml function and followed by the optim.pml function with optNni = T, performing Jukes-Cantor optimization. The resulting ML trees, in some cases, were visualized as phylograms using phangorn′s internal functionality and in others as circular tree structures using the ggtree and dendextend R packages^83,84^.

LD was assessed for all variants in the exonic regions of G1 alleles merged with matched 3′ UTR haplotig sequences. Variants with low minor allele frequencies (MAF) were removed by requiring a MAF > 0.1. The extent of LD was assessed by computing the D value for all remaining variant pairs, and the correlation coefficient was computed from these D values using standard formulae for LD analysis^85^. Variants were then hierarchically clustered using the R hclust function with a distance matrix produced from these correlations as input. After visual examination of the tree generated from this clustering, correlated groups of variants were split using the cutree function with a height of 0.35, implicitly requiring a minimum correlation of 0.9 within groups. Resulting in groups of size two that were not in complete LD, being further split into individual groups of size one.

Statistical tests for association with protection outcome for individual variants were performed using Fisher′s Exact tests (fisher.test R function) followed by Benjamini–Hochberg (BH) multiple hypothesis testing correction for FDR control. 2 × 2 contingency tables were constructed by comparing animals with homozygous major variants against heterozygotes and those with homozygous minor variants stratified across protection outcomes (protected, not protected). These same statistical tests for association were applied to correlated groups of variants followed by BH FDR control. Only animals in groups O, S, and X were used for all tests for association with protection outcome. This same procedure was performed for statistical tests for association with the vaccine group, where all animals from groups O, S, X, and E were used. Fisher′s exact tests were also to test these associations.

### Expression analysis of *Mamu-E* loci

The expression of alleles from different groups was performed by generating STAR alignment indexes tailored to each animal. The MHC Class I/II BAC reference described above was included with *Mamu-E* masked out. Trimmed alleles from each group were included as additional contigs. Alleles from the different G2 subgroups were kept as separate contigs when present in the same animal. G2 alleles containing an LTR in their promoter region were left untrimmed, and the LTR was annotated using Dfam release 3.1^53^ with the organism set to Homo Sapiens. When two allele sequences were present from a group, a reference was chosen, and the other allele was represented in a VCF file as an alternative. This file was generated by aligning the two allele sequences using the needle tool from the EMBOSS Suite^86^ and extracting positions harboring SNPs. The needle was run using the parameters -endopen 0, -endextend 0, -gapopen 100, -gapextend 0. The reference alleles included in the index were annotated by aligning the canonical *Mamu-E1* isoform sequence with exon 8 removed (not included in the allele sequences) using exonerate^87^. For G3 alleles, which did not contain exon 6, *Mamu-E7* was used (skips exon 6). Since no stop codon was expected in these annotations, we used the exonerate est2genome model with –showtargetgff yes to extract annotation records for alleles.

Raw whole blood mRNA-seq reads were aligned using STAR 2.7.7a^67^ in WASP mode^88^ with the same base parameters used above when phasing haplotypes from mRNA-seq. WASP mode removes the allele-specific bias that might be introduced by selecting one allele as the reference. WASP mode was run by adding the additional parameters: –outSAMattributes NH HI AS nM vA vG, –varVCFfile [VCF file], –waspOutputMode SAMtag. When a VCF file was not present (i.e., no allele group had >1 allele), only –outSAMattributes NH HI AS nM was added as an additional parameter.

Relative expression of allele groups was determined by using the gene counts produced by STAR for each group. To examine the relative expression of alleles within a group (where applicable), we extracted reads properly paired and uniquely mapped to the *Mamu-E* allele contigs using samtools with -q 255 and -f 0×2 parameters and using the bedtools intersect function^77^, selecting those with the vA flag set. Reads with vA set to i:1 and i:2 were assigned to the reference allele and alternate allele, respectively. Reads with vA set to i:0 were common to both alleles. Reads with vA set to any other values (i:1,2 or i:3) or with the WASP flag turned on (i.e., set to a value other than i:1) were removed from the analysis.

### Relative *Mamu-E* isoform expression analysis

Isoform expression analysis was performed similarly to the *Mamu-E* locus analysis. To include *Mamu-E* isoform annotations spanning the 3′ UTR, G1 alleles merged with the 3′ UTR haplotig sequence were used in place of the trimmed G1 alleles. Annotations for all *Mamu-E* isoforms were generated using exonerate^87^ run with the cdna2genome model. In cases where G2 and G3 alleles were present in the animal, they were still included as separate contigs, as described above. *Mamu-E* transcriptome alignments were then generated when aligning to this index by adding “TranscriptomeSAM” to the –quantMode parameter field. Relative isoform abundances were then calculated using salmon^89^ with the default VBEM algorithm and 25 bootstraps. Final isoform relative abundances were calculated by using the mean of the bootstrap estimates.

### *Mamu-E* duplication and G1 allele association analysis

Statistical tests were separately performed to assess the significance of the association between either *Mamu-E* duplications or G1 allele subgroups with either protection outcome or vaccine group (four tests in total). When assessing *Mamu-E* duplication associations, animals with either a G2 or G3 allele were grouped together, thus forming two groups of animals (G1, G1 + G2/G3). For association with protection outcome, only animals from the O, S, and X groups were used, and animals were stratified by protection outcome (protected, not protected). In cases with 2 × 2 contingency tables, Fisher′s Exact tests were used to assess significance in R (fisher.test function).

### Statistics and reproducibility

Statistical tests are described as appropriate separately in the following Methods sections: “Whole blood mRNA-seq and Mamu-E haplotype phasing,” “Mamu-E phylogenetic and genetic analysis,” and “Mamu-E duplication and G1 allele association analysis”.

### Reporting summary

Further information on research design is available in the Nature Research Reporting Summary linked to this article.

## Supplementary information

Supplementary Information

Description of Additional Supplementary Data

Supplementary Data 1

Reporting Summary-New

## Data Availability

Source data for Figs. 3–5 and Supplementary Figs. 1, 3–5, and 7–9 are available in Supplementary Data 1. Sequence FASTA and annotation GTF files for RM and human *MHC-E* isoforms, Sanger sequencing data, and *Mamu-E* genotyping data were deposited to Zenodo (doi: 10.5281/zenodo.7107936). *Mamu-E* allele sequences were deposited to GenBank under accession numbers MT221257 through MT221434. Transcriptomic data for vaccine groups O, S, and X are available in the Gene Expression Omnibus (GEO) https://www.ncbi.nlm.nih.gov/geo/ under accession number GSE160562. Transcriptomic data for vaccine group E is available under BioProject accession number PRJNA825389 in the NCBI BioProject database (https://www.ncbi.nlm.nih.gov/bioproject/). All other data are available from D.E.G. and X.P. upon request.

## Code availability

All code used for post-processing PacBio LAA data were deposited to Zenodo along with instructions for use.
