# Supplementary material for: Alternative splicing and genetic variation of mhc-e: implications for rhesus cytomegalovirus-based vaccines
Source: Commun Biol. 2022 Dec 19;5:1387. doi: 10.1038/s42003-022-04344-2 (PMC9762870; doi:10.1038/s42003-022-04344-2)
Supplement: Supplementary file 1 — Supplementary Information [file 42003_2022_4344_MOESM1_ESM.pdf]

ALTERNATIVE SPLICING AND GENETIC VARIATION OF MHC-E: IMPLICATIONS FOR RHESUS CYTOMEGALOVIRUS-BASED VACCINES

SUPPLEMENTARY INFORMATION

Hayden N. Brochu[1,2], Ruihan Wang[3], Tammy S. Tollison[1], Chul-Woo Pyo[3], Alexander Thomas[3], Elizabeth Tseng[4], Lynn Law[5,6], Louis J. Picker[7], Michael Gale, Jr.[5,6,8], Daniel E. Geraghty[3,*], and Xinxia Peng[1,2,9,*]

[1]Department of Molecular Biomedical Sciences, North Carolina State University College of Veterinary Medicine, Raleigh, NC 27607, USA; [2]Bioinformatics Graduate Program, North Carolina State University, Raleigh, NC 27695, USA; [3]Clinical Research Division, Fred Hutchinson Cancer Research Center, Seattle, WA 98109, USA; [4]Pacific Biosciences, Menlo Park, CA 94025, USA; [5]Department of Immunology, University of Washington, Seattle, WA, USA; [6]Center for Innate Immunity and Immune Diseases, University of Washington, Seattle, WA, USA; [7]Vaccine and Gene Therapy Institute, Oregon Health & Science University, Beaverton, OR 97006, USA; [8]Washington National Primate Research Center, University of Washington, Seattle, WA, USA; [9]Bioinformatics Research Center, North Carolina State University, Raleigh, NC 27695, USA; *Corresponding authors

*Address correspondence requests to Dr. Daniel E. Geraghty (geraghty@fredhutch.org) and Dr. Xinxia Peng (xpeng5@ncsu.edu).

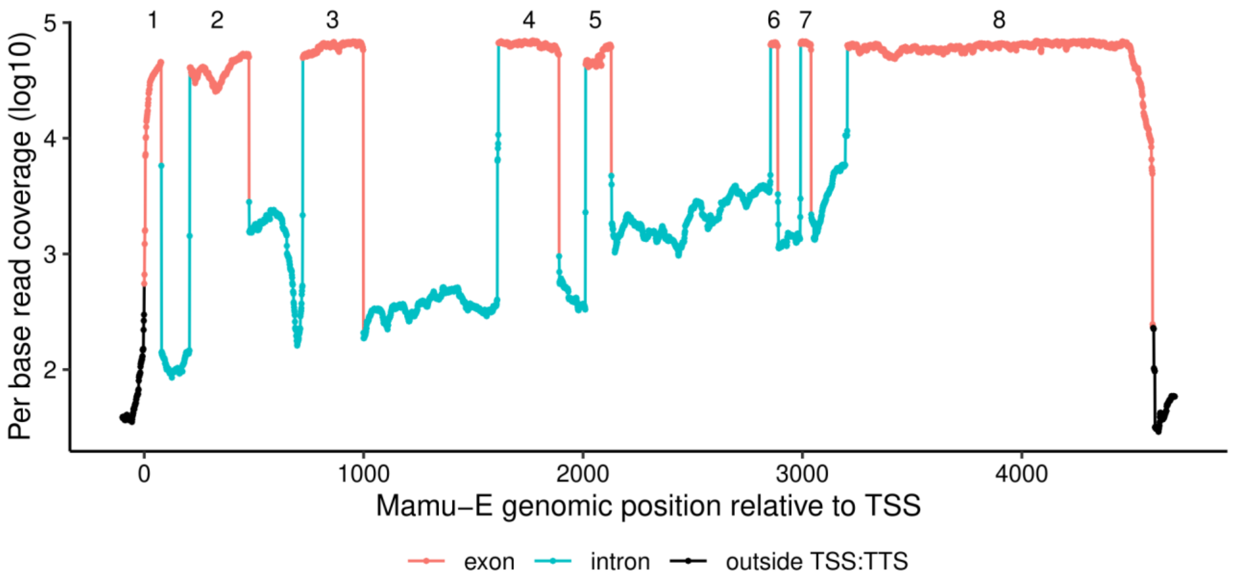

Supplementary Figure 1. mRNA-seq coverage of *Mamu-E* locus. Pre-challenge whole blood samples from 60 RM were aligned to the *Mamu-E* locus, alignments were pooled for each animal, and the mean per base coverage (log10) across animals is shown. Regions of the locus are shown in red (exonic), blue (intronic), and black (100bp upstream of the TSS and downstream of the TTS). Positions are shown relative to the TTS and the canonical exon numbers are shown at the top above the coverage profile. On average, 50,000 reads were recovered per sample, and when pooling all nine pre-challenge samples for each animal ~10,000 per base coverage was observed in all exonic regions (red).

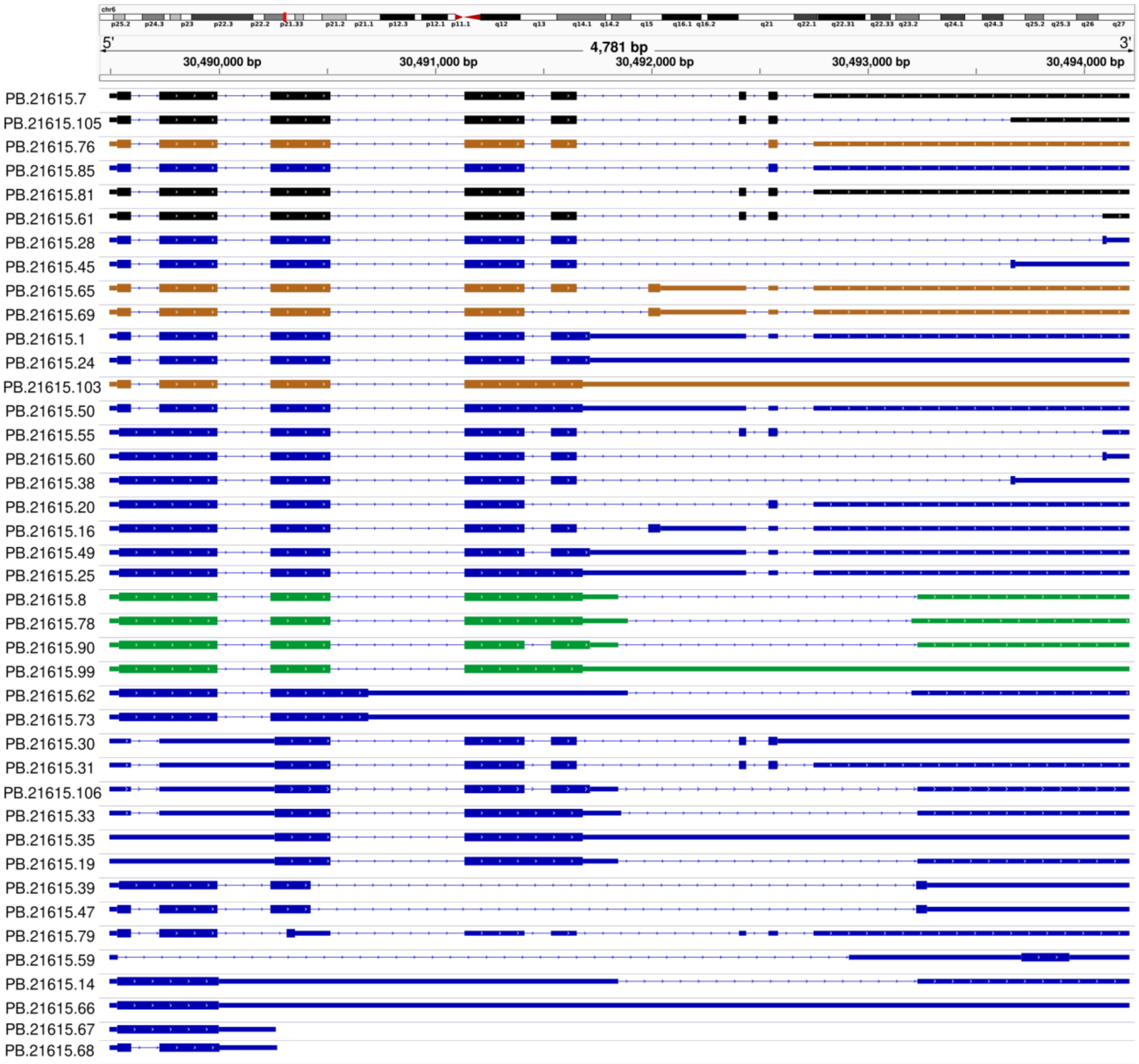

Supplementary Figure 2. *HLA-E* alternative splicing. 45 *HLA-E* isoforms generated by Iso-Seq are shown by their genomic alignments. Exons are shown as rectangles with lines representing introns removed by splicing. Thicker segments represent the coding regions of transcripts. Transcript are shown from 5’ (left) to 3’ (right). Black transcripts had complete matches to *Mamu-E* isoforms. Brown isoforms had complete matches to partial *Mamu-E* isoforms and were used to infer their 5’ ends. Green isoforms had novel splicing junctions also identified in *Mamu-E* isoforms and were supporting evidence for inferring complete 5’ ends. Many isoforms in blue contain splice junctions present in *Mamu-E* isoforms and have additional splicing complexity.

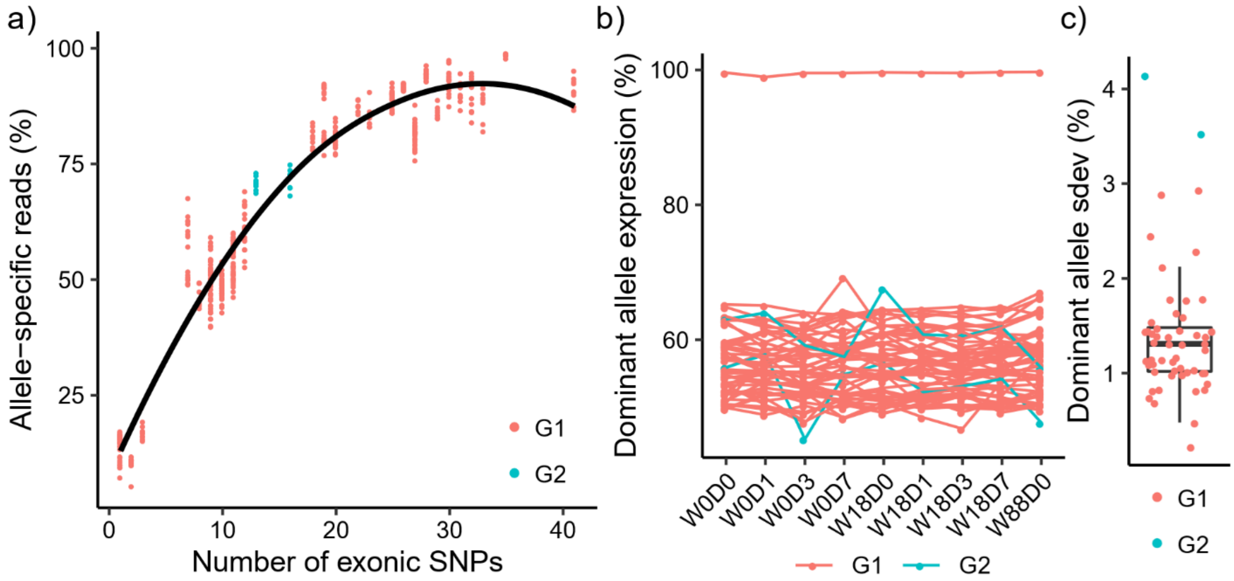

Supplementary Figure 3. Analysis of allele groups with 2 alleles expressed within each animal (n = 54, G3 not included as it is monomorphic). Each locus (G1 and G2) is analyzed separately. Two animals had two G2 alleles, shown in blue in all three panels. **a)** Relationship between the allelic polymorphism at a *Mamu-E* locus and the proportion (%) of reads uniquely assigned to an allele, where each data point (n = 9 per animal) represents a sample from an animal. A loess curve is shown in black. **b)** Expression of the dominant allele at the locus (i.e. allele with higher expression of the pair) during the pre-challenge phases. The animal with ~100% expression from one allele has a frameshift caused by an insertion in its second allele. **c)** Standard deviation (sdev) of dominantly expressed alleles over the pre-challenge phase. Alleles derived from the G1 and G2 groups are colored red and blue, respectively. Boxplots are defined by quartiles (upper, median, lower) with whiskers extending to data points up to 1.5 times the interquartile range.

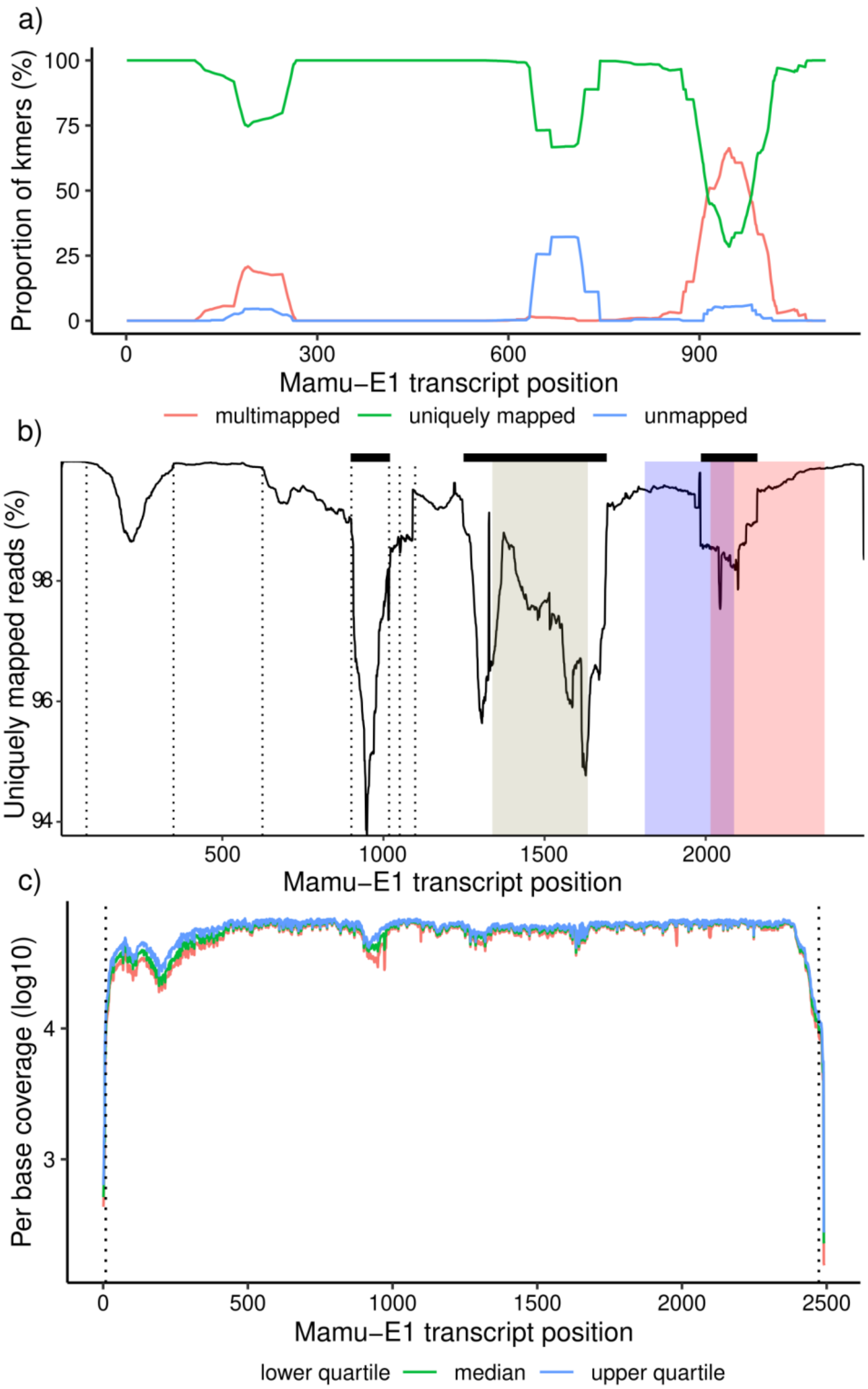

Supplementary Figure 4. Limitations of *Mamu-E* haplotype phasing using mRNA-seq reads. **a)** Proportion of kmers derived from *Mamu-E* alleles that uniquely map (green), multimap (red), and don't map (blue) to the reference *Mamu-E1* transcript (exons 1-7). **b)** Proportion of reads that uniquely map to various regions of the full *Mamu-E1* transcript. Exons are delimited by vertical dotted lines, locations of transposable elements are colored in (khaki = AluY, blue = MSTC, red = MLT-int, and purple = overlap of MSTC and MLT-int). Regions with black bars overhead have low proportions of uniquely mapped reads and are excluded from haplotype phasing analysis. **c)** Per base coverage of the *Mamu-E1* transcript (log10) with lines colored by quartile. Vertical dotted lines demarcate 3' and 5' ends with coverage too low for haplotype phasing analysis.

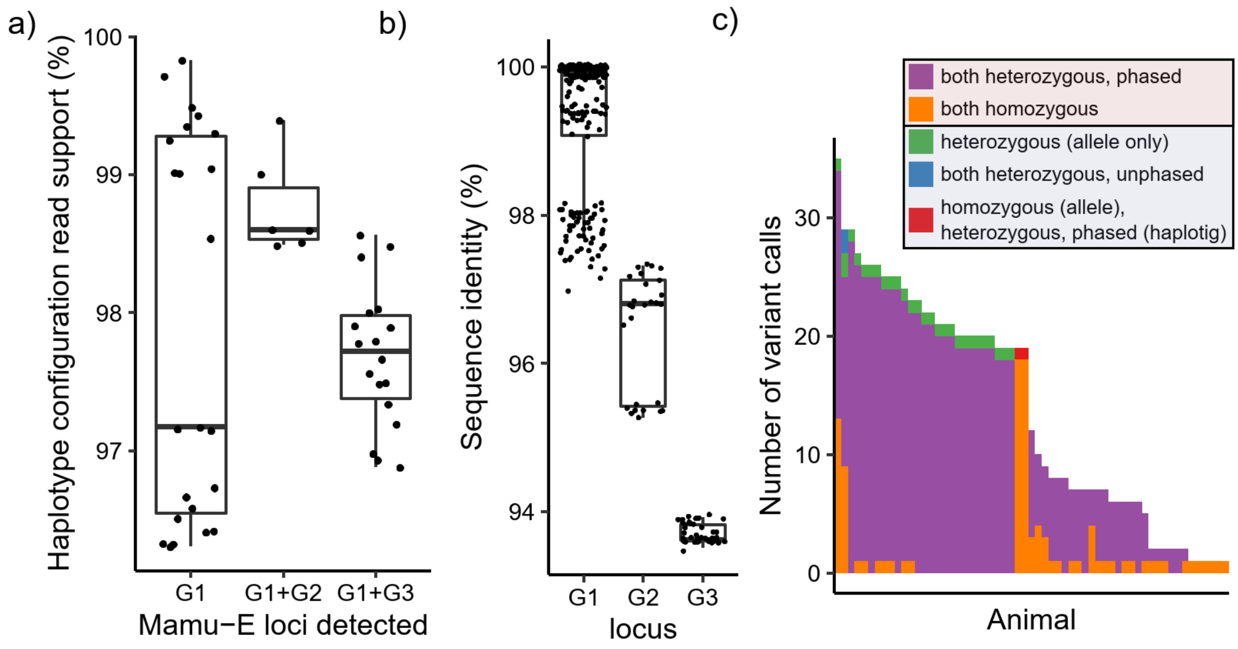

Supplementary Figure 5. mRNA-seq haplotype phasing and integration with allele sequences. **a)** The proportion of reads support variant connections in the haplotype configuration, where each data point represents an animal with at least one heterozygous variant (G1: n = 23, G1+G2: n = 6, G1+G3: n = 18). Animals are stratified based on the presence/absence of additional alleles from the G2 and G3 allele groups. **b)** Sequence identity between all haplotigs and all alleles from each allele group within the same animal (G1: n = 221, G2: n = 27, G3: n = 40). **c)** Comparison of variant phasing for each animal (n = 59), where alleles are matched to haplotigs. Colors of bars indicate the type of match, where purple and orange are concordant heterozygous and homozygous variants, respectively. In **a-c**, allelic indel variation is excluded from comparisons. In **(a-b)**, boxplots are defined by quartiles (upper, median, lower) with whiskers extending to data points up to 1.5 times the interquartile range.

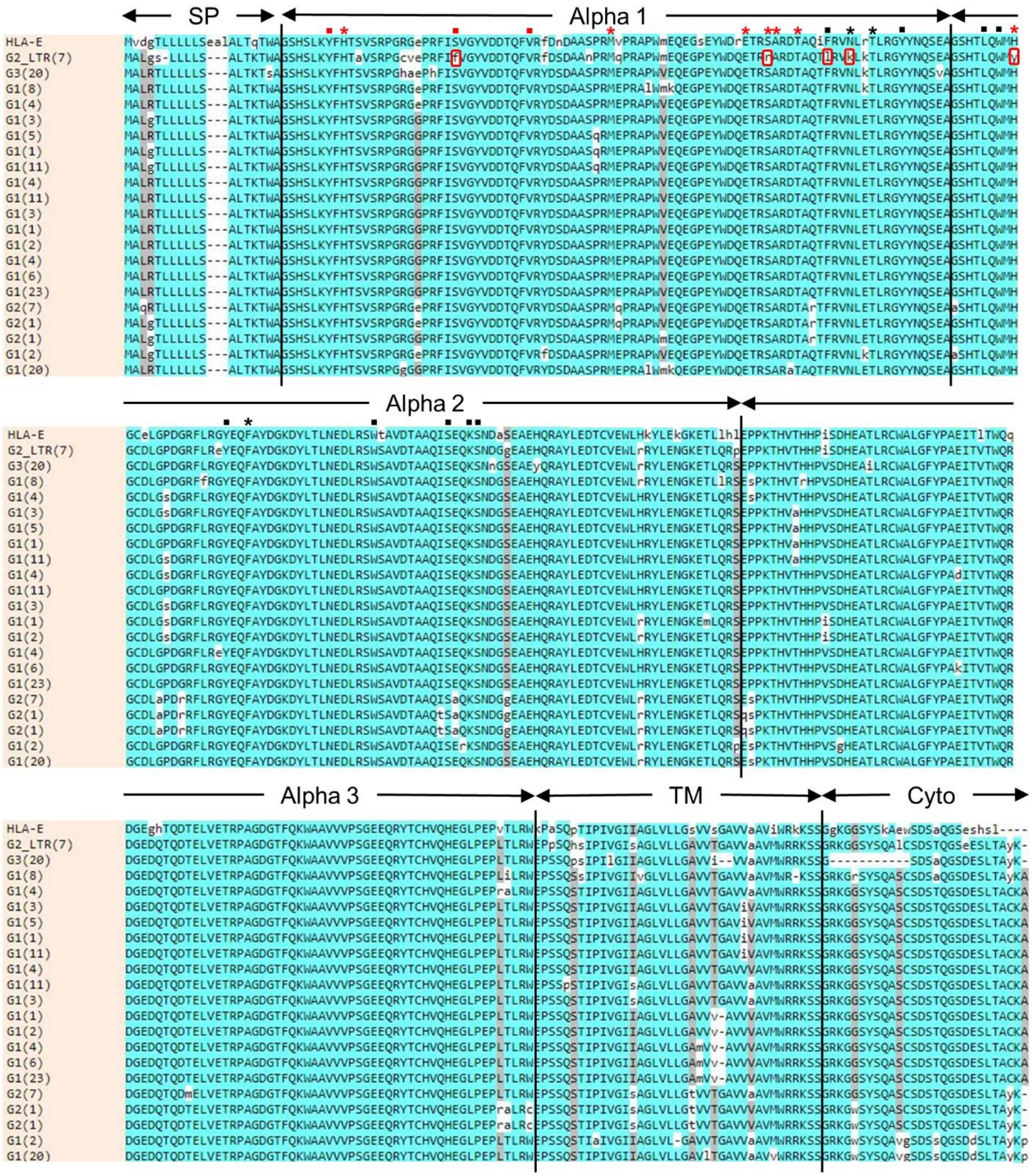

Supplementary Figure 6. Multiple sequence alignment of all Mamu-E protein sequences and HLA-E. The number of alleles supporting each protein sequence is shown in parentheses next to the allele designation on the left. All protein domains are demarcated by vertical bars and annotated above the alignments. B and F pocket residues are denoted by red and black symbols above the alignment. Those that are consider key residues are denoted by asterisks while all other s are denoted by squares. With the exception of G2_LTR alleles, all B and F pocket residues are conserved between HLA-E and Mamu-E. G2_LTR polymorphisms in at these sites are denoted by red boxes.

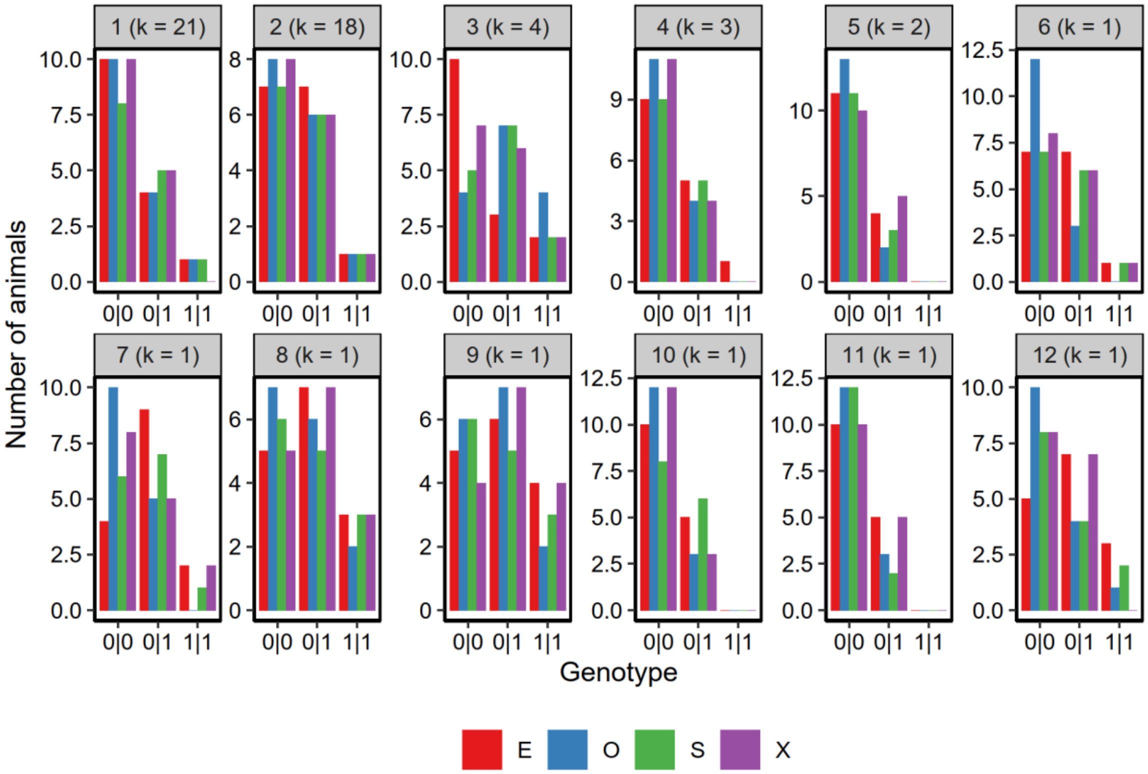

Supplementary Figure 7. Number of animals from each vaccine group stratified by genotype for all variant clusters. Vaccine groups E, O, S, and X are colored red, blue, green, and purple, respectively. Variant clusters are ordered and labeled by the number of variants included in the cluster, beginning with largest in the top left.

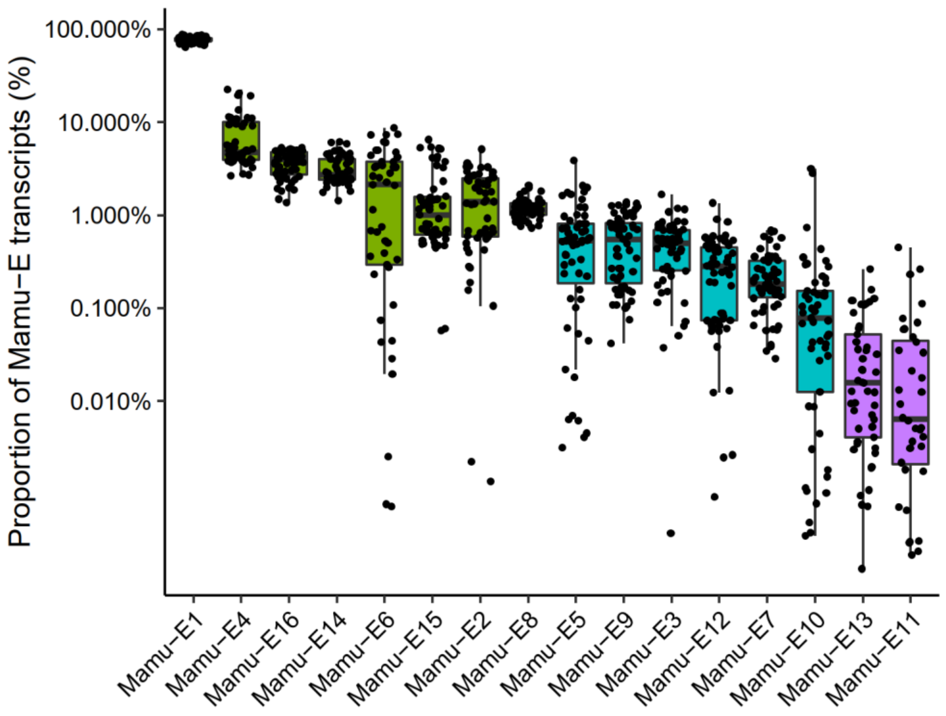

Supplementary Figure 8. Proportion of *Mamu-E* transcripts derived from each isoform. Isoforms are colored by strata defined by their median proportions, beginning with *Mamu-E1* (>10%), followed by green (1 to 10%, n = 7), blue (0.1 to 1%, n = 6), and purple (<0.1%, n = 2) isoform boxplots. Each data point represents the proportion of *Mamu-E* transcripts for an animal averaged over all pre-challenge time points (n = 59 animals). Boxplots are defined by quartiles (upper, median, lower) with whiskers extending to data points up to 1.5 times the interquartile range.

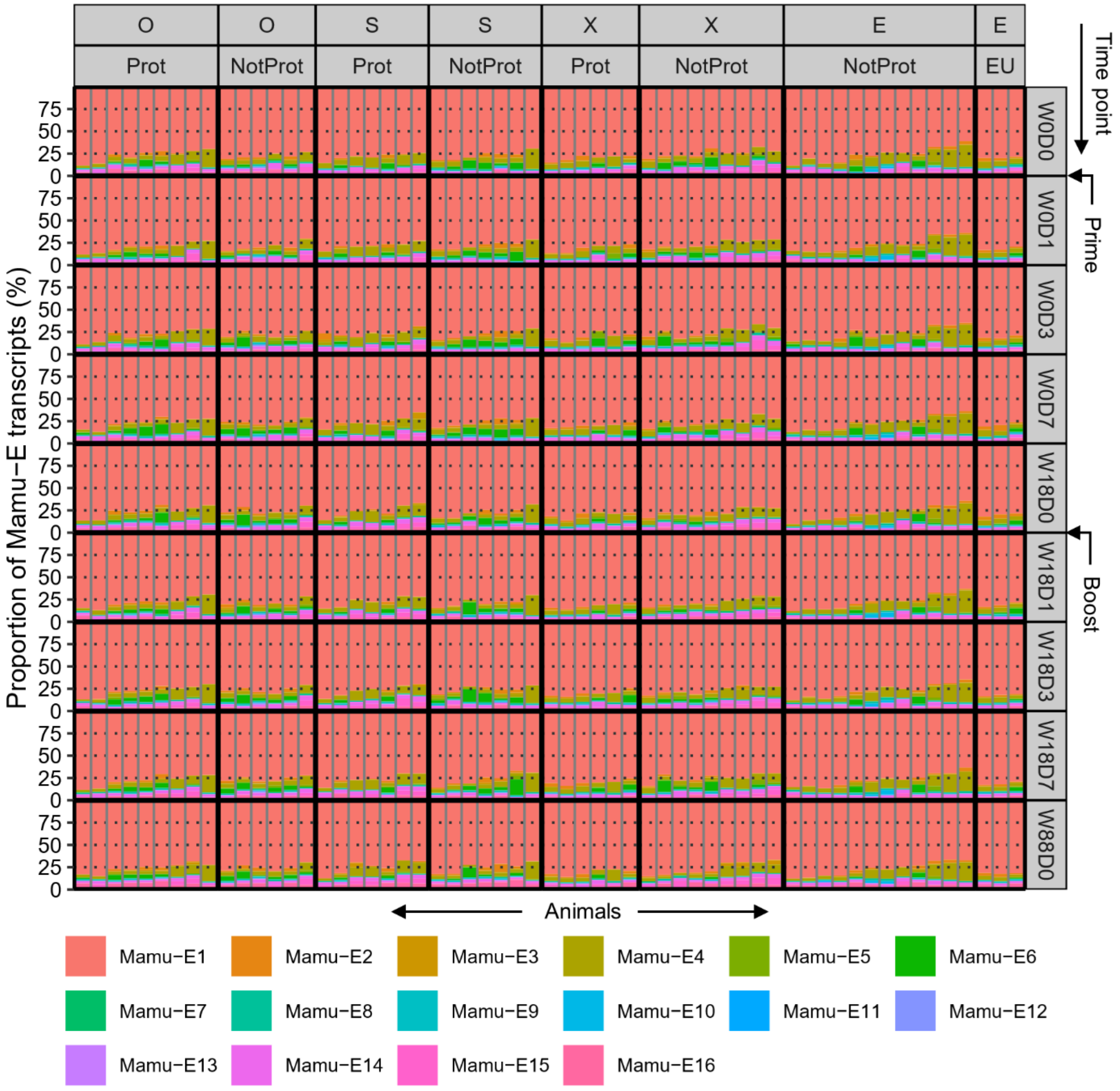

Supplementary Figure 9. Relative *Mamu-E* isoform abundance throughout the pre-challenge phase of a RhCMV/SIV vaccine study. Each column is a single animal, where animals are separated by vaccine group and protection outcome (Prot = protected, NotProt = not protected, EU = exposed-uninfected). Relative proportions of isoforms are shown as stacked bar plots with different colors representing isoforms. Each time point in the pre-challenge phase is displayed as a row, beginning with W0D0 at the top.

Supplementary Table 1. PacBio Iso-Seq of four tissues: lymph node, peripheral blood mononuclear cells, rectal biopsy, and whole blood. CCS reads are labeled as full if they contain 5' primer, 3' primer, and polyA; otherwise, they are labeled partial.

Tissue	Type of CCS read	Number of CCS reads
**Lymph Node**	Full	234,348
	Partial	503,492
**Peripheral Blood Mononuclear Cells**	Full	183,174
	Partial	401,001
**Rectal Biopsy**	Full	261,011
	Partial	535,317
**Whole Blood**	Full	243,466
	Partial	442,883
**Total**	Full	921,999
	Partial	1,882,693

Supplementary Table 2. CCS read support for *Mamu-E* isoforms detected by PacBio Iso-Seq. CCS read counts are shown separately for whole blood (WB), peripheral blood mononuclear cells (PBMC), lymph node (LN), and rectal biopsy (RB). *Mamu-E8* and *Mamu-E9* are two isoforms with the same CCS read support but that had different 5' ends inferred from matched *HLA-E* isoforms.

Isoform	WB	PBMC	LN	Rect	Total
Mamu-E1	45	24	21	2	92
Mamu-E2	4	4	5	0	13
Mamu-E3	1	0	1	0	2
Mamu-E4	1	0	0	0	1
Mamu-E5	1	0	0	0	1
Mamu-E6	3	1	3	0	7
Mamu-E7	0	0	0	1	1
Mamu-E8, Mamu-E9	0	0	1	0	1
Mamu-E10	0	1	0	0	1
Mamu-E12	0	1	0	0	1
Mamu-E14	1	0	0	0	1
Mamu-E16	0	1	0	0	1
**Total**	56	32	32	3	123

Supplementary Table 3. Protein domain and 3' untranslated region (UTR) configurations of *HLA-E* isoforms. The inclusion, exclusion, or alteration of protein domains is shown for each *HLA-E* isoform, where deviations from the canonical isoform (PB.25615.7) are shown in bold. *HLA-E* isoforms containing all three alpha domains are shown in bold. Alternate domains or 3' UTRs are given unique number assignments. All domains correspond to a single exon with the exception of the cytoplasmic domain, which is derived from two exons. Cytoplasmic domains are labeled as partially skipped when only one of its two exons is skipped.

**Isoform**	**Domains**						**3' UTR**
	**Signal peptide**	**α_1**	**α_2**	**α_3**	**Transmembrane**	**Cytoplasmic**	
**PB.21615.7**	Yes	Yes	Yes	Yes	Yes	Yes	Yes
**PB.21615.105**	Yes	Yes	Yes	Yes	Yes	Yes	**Alternate-1**
**PB.21615.76**	Yes	Yes	Yes	Yes	Yes	**Partially skipped**	Yes
**PB.21615.85**	Yes	Yes	Yes	Yes	**No**	**Partially skipped**	Yes
**PB.21615.81**	Yes	Yes	Yes	Yes	**No**	Yes	Yes
**PB.21615.61**	Yes	Yes	Yes	Yes	Yes	Yes	**Alternate-2**
**PB.21615.28**	Yes	Yes	Yes	Yes	Yes	**No**	**Alternate-2**
**PB.21615.45**	Yes	Yes	Yes	Yes	Yes	**No**	**Alternate-1**
**PB.21615.65**	Yes	Yes	Yes	Yes	Yes	**No**	Yes
**PB.21615.69**	Yes	Yes	Yes	Yes	No	**No**	Yes
**PB.21615.1**	Yes	Yes	Yes	Yes	Yes	**No**	Yes
**PB.21615.24**	Yes	Yes	Yes	Yes	Yes	**No**	Yes
**PB.21615.103**	Yes	Yes	Yes	Yes	Yes	**No**	Yes
**PB.21615.50**	Yes	Yes	Yes	Yes	Yes	**No**	Yes
**PB.21615.55**	**Alternate-1**	Yes	Yes	Yes	Yes	Yes	**Alternate-2**
**PB.21615.60**	**Alternate-1**	Yes	Yes	Yes	Yes	**No**	**Alternate-2**
**PB.21615.38**	**Alternate-1**	Yes	Yes	Yes	Yes	**No**	**Alternate-1**
**PB.21615.20**	**Alternate-1**	Yes	Yes	Yes	**No**	**Partially skipped**	Yes
**PB.21615.16**	**Alternate-1**	Yes	Yes	Yes	Yes	**No**	Yes
**PB.21615.49**	**Alternate-1**	Yes	Yes	Yes	Yes	**No**	Yes
**PB.21615.25**	**Alternate-1**	Yes	Yes	Yes	**No**	**No**	Yes
**PB.21615.8**	**Alternate-1**	Yes	Yes	Yes	**No**	**No**	**Alternate-3**
**PB.21615.78**	**Alternate-1**	Yes	Yes	Yes	**No**	**No**	**Alternate-4**
**PB.21615.90**	**Alternate-1**	Yes	Yes	Yes	Yes	**No**	**Alternate-3**
**PB.21615.99**	**Alternate-1**	Yes	Yes	Yes	**No**	**No**	Yes
PB.21615.62	**Alternate-1**	Yes	Yes	**No**	**No**	**No**	**Alternate-4**
PB.21615.73	**Alternate-1**	Yes	Yes	**No**	**No**	**No**	Yes
PB.21615.30	**No**	**No**	**3' partial**	Yes	Yes	Yes	Yes
PB.21615.31	**No**	**No**	**3' partial**	Yes	Yes	Yes	Yes
PB.21615.106	**No**	**No**	**3' partial**	Yes	Yes	**No**	**Alternate-4**
PB.21615.33	**No**	**No**	**3' partial**	Yes	**No**	**No**	**Alternate-4**
PB.21615.35	**No**	**No**	**3' partial**	Yes	**No**	**No**	Yes
PB.21615.19	**No**	**No**	**3' partial**	Yes	**No**	**No**	**Alternate-4**
PB.21615.39	**Alternate-1**	Yes	**5' partial**	**No**	**No**	**No**	**Alternate-4**
PB.21615.47	Yes	Yes	**5' partial**	**No**	**No**	**No**	**Alternate-4**
PB.21615.79	Yes	Yes	**No**	**No**	**No**	**No**	Yes
PB.21615.59	**No**	**No**	**No**	**No**	**No**	**No**	**Alternate-5**
PB.21615.14	**Alternate-1**	Yes	**No**	**No**	**No**	**No**	**Alternate-4**
PB.21615.66	**Alternate-1**	Yes	**No**	**No**	**No**	**No**	Yes
PB.21615.67	**Alternate-1**	Yes	**No**	**No**	**No**	**No**	**No**
PB.21615.68	Yes	Yes	**No**	**No**	**No**	**No**	**No**

Supplementary Table 4. Primers used for PCR and Sanger sequencing. Primers are named whether they are used as forward (FWD) or reverse (REV) primers in reactions. Target isoforms and isoform regions are shown for each primer. The first primer shown (FWD_Exon1) is common to all PCR reactions and targets the 5' UTR. The second (REV_POS) is a positive control targeting a splice junction common among the *Mamu-E* isoforms. The last two primers shown (FWD_Sanger_EX4, REV_Sanger_EX4) were used only for Sanger sequencing when the expected amplicon exceeded the Sanger size limitations (Methods).

**Primer name**	**Primer sequence**	**Isoform/region target**
FWD_Exon1	AATGGGGTCATGGCACTACG	5' UTR
REV_POS	GGAAGACGGCTCCCATCTCA	Canonical exon4/exon5 splice junction
REV_PB.25.9	ACAGCAACCACGCCATT	*Mamu-E10*
REV_PB.25.8+10	GATGACACTCTGAACAGCCCA	*Mamu-E4*, *Mamu-E10*
REV_PB.25.11	ATTCTCCTGGGAGGCAGA	*Mamu-E12*
REV_PB.25.12	GGTACTGTCGCTACCTGAGC	*Mamu-E11*
REV_PB.25.13	TCTGTCCTTCATTGTCACAGGT	*Mamu-E14*
REV_PB.25.14_1	AACCCCATCTCTACCCATCTCA	*Mamu-E8*
REV_PB.25.14_2	ACCCCATCTCTACTGAGCTCTT	*Mamu-E9*
FWD_Sanger_EX4	CTGATCATGAGGCCACCCTG	Canonical exon4 (5' end)
REV_Sanger_EX4	CGCTGCCAGGTCACTGT	Canonical exon4 (3' end)

Supplementary Table 5. PacBio LAA alleles recovered for 59 RhCMV/SIV vaccinated animals. Animals are grouped by group and challenge outcome and allele(s) from each group (G1, G2, G2_LTR, G3) are shown.

**Animal**	**Group**	**Challenge Outcome**	**PacBio LAA alleles**		
			**G1**	**G2, G2_LTR#**	**G3**
**Rh28808**	O	Protected	RH28808-C1_E*02:01:02, RH28808-C2_E*02:04		RH28808-C3_E*02:13V-short
**Rh28885**			RH28885-C1_E*02:11, RH28885-C2_E*02:03		
**Rh28890**			RH28890-C1_E*02:03V, RH28890-C2_E*02:02	RH28890-C3_E*02:12:01V, RH28890C-4_E*02:12/28V	
**Rh28949**			RH28949-C1_E*02:10, RH28949-C2_E*02:11		
**Rh29212**			RH29212-C1_E*02:01:02, RH29212-C2_E*02:01:02V		
**Rh29215**			RH29215-C3_E*02:04, RH29215-C5_E*02:11		RH29215-C1_E*02:13V-short
**Rh29482**			RH29482-C1_E*02:11, RH29482-C2_E*02:11		
**Rh29483**			RH29483-C2_E*02:04, RH29483-C3_E*02:03V		RH29483-C1_E*02:13V-short
**Rh30407**			RH30407-C1_E*02:04		RH30407-C2_E*02:13V-short
**Rh28724**		Not Protected	Rh28724-c1_E*02:11, Rh28724-c2_E*02:03		
**Rh28810**			RH28810-C1_E*02:11, RH28810-C2_E*02:18		
**Rh29014**			RH29014-C1_E*02:11, RH29014-C2_E*02:11	RH29014-C3_E*02:20V#	
**Rh29306**			RH29306-C1_E*02:04, RH29306-C2_E*02:11		RH29306-C3_E*02:13V-short
**Rh29528**			RH29528-C1_E*02:11, RH29528-C2_E*02:11	RH29528-C3_E*02:20V#	
**Rh30400**			RH30400-C1_E*02:11V, RH30400-C5_E*02:02	RH30400-C9_E*02:20V#, RH30400-C4_E*02:12:01V	
**Rh29208**	S	Protected	RH29208-C1_E*02:03, RH29208-C4_E*02:02	RH29208-C5_E*02:12:01V, RH29208-C3_E*02:16	
**Rh29211**			RH29211-C1_E*02:11V, RH29211-C2_E*02:03		
**Rh29299**			RH29299-C2_E*02:07, RH29299-C3_E*02:04		RH29299-C1_E*02:13V-short
**Rh29341**			RH29341-C2_E*02:04, RH29341-C3_E*02:11		RH29341-C1_E*02:13V-short
**Rh29515**			RH29515-C1_E*02:04, RH29515-C3_E*02:11		RH29515-C2_E*02:13V-short
**Rh29558**			RH29558-C1_E*02:01:02, RH29558-C2_E*02:10		
**Rh30028**			RH30028-C1_E*02:01:02, RH30028-C2_E*02:04		RH30028-C3_E*02:13V-short
**Rh28819**		Not Protected	RH28819-C1_E*02:03, RH28819-C2_E*02:09V		
**Rh28835**			RH28835-C1_E*02:11, RH28835-C3_E*02:10/11V	RH28835-C2_E*02:30V#	
**Rh29484**			RH29484-C1_E*02:14, RH29484-C2_E*02:10		
**Rh29511**			RH29511-C2_E*02:03, RH29511-C3_E*02:04		RH29511-C1_E*02:13V-short
**Rh29523**			RH29523-C1_E*02:11, RH29523-C2_E*02:11	RH29523-C4_E*02:20V#	
**Rh30412**			RH30412-C1_E*02:11, RH30412-C2_E*02:03		
**Rh30426**			RH30426-C1_E*02:04, RH30426-C2_E*02:05V		RH30426-C3_E*02:13V-short
**Rh28809**	X	Protected	RH28809-C1_E*02:04, RH28809-C3_E*02:02		RH28809-C2_E*02:13V-short
**Rh28930**			RH28930-C1_E*02:10, RH28930-C2_E*02:11		
**Rh29292**			RH29292-C1_E*02:11, RH29292-C3_E*02:02	RH29292-C4_E*02:12:01V	

Supplementary Table 5 (continued).

**Rh29300**	X	Protected	RH29300-C1_E*02:01:02, RH29300-C2_E*02:18		
**Rh29522**			RH29522-C1_E*02:03, RH29522-C2_E*02:18		
**Rh30445**			RH30445-C1_E*02:01:02, RH30445-C2_E*02:03V		
**Rh28899**		Not Protected	RH28899-C1_E*02:07, RH28899-C2_E*02:03V		
**Rh29084**			RH29084-C1_E*02:01:02, RH29084-C2_E*02:04		RH29084-C3_E*02:13V-short
**Rh29214**			RH29214-C1_E*02:11, RH29214-C2_E*02:14		
**Rh29479**			RH29479-C1_E*02:04, RH29479-C2_E*02:07		RH29479-C3_E*02:13V-short
**Rh29535**			RH29535-C1_E*02:04, RH29535-C2_E*02:11		RH29535-C3_E*02:13V-short
**Rh29548**			RH29548-C1_E*02:02, RH29548-C1_E*02:11	RH29548-C3_E*02:12:01V	
**Rh30025**			RH30025-C1_E*02:11V, RH30025-C2_E*02:11	RH30025-C3_E*02:20V#	
**Rh30427**			RH30427-C1_E*02:03, RH30427-C2_E*02:11		
**Rh30434**			RH30434-C1_E*02:04, RH30434-C2_E*02:11		RH30434-C3_E*02:13V-short
**Rh28828**	E	Not Protected	RH28828-C1_E*02:01:02, RH28828-C2_E*02:05V		
**Rh28833**			RH28833-C1_E*02:04, RH28833-C2_E*02:18		RH28833-C3_E*02:13V-short
**Rh28918**			RH28918-C1_E*02:03V, RH28918-C2_E*02:02		
**Rh28938**			RH28938-C1_E*02:04, RH28938-C2_E*02:09		RH28938-C3_E*02:13V-short
**Rh29116**			RH29116-C1_E*02:10, RH29116-C2_E*02:11		
**Rh29301**			RH29301-C1_E*02:04, RH29301-C3_E*02:14		RH29301-C2_E*02:13V-short
**Rh29514**			RH29514-C1_E*02:11	RH29514-C2_E*02:20V#	
**Rh29569**			RH29569-C1_E*02:01:02, RH29569-C4_E*02:03, RH29569-C5_E*02:02		
**Rh29807**			RH29807-C1_E*02:01:02, RH29807-C2_E*02:03		
**Rh30410**			RH30410-C1_E*02:03V, RH30410-C2_E*02:03V		
**Rh30418**			RH30418-C1_E*02:03V, RH30418-C2_E*02:02	RH30418-C4_E*02:16V, RH30418-C3_E*02:12:01V	
**Rh30429**			RH30429-C1_E*02:04		RH30429-C2_E*02:13V-short
**Rh29296**		Exposed-Uninfected	RH29296-C1_E*02:01:02, RH29296-C2_E*02:09		
**Rh29305**			RH29305-C1_E*02:14, RH29305-C2_E*02:03		
**Rh29560**			RH29560-C1_E*02:03V, RH29560-C2_E*02:10		

Supplementary Table 6. Fosmid sequences of select animals. Alleles recovered by PacBio Long Amplicon Analysis (LAA) are shown alongside assembled fosmid sequence(s) recovered using additional PacBio sequencing.

Animal	PacBio LAA alleles	Fosmid sequence(s)
Rh28808	E*02:01:02, E*02:04, E*02:13V-short	E*02:13V-short/E*02:04
Rh28809	E*02:04, E*02:02, E*02:13V-short	E*02:13V-short
Rh28835	E*02:11, E*02:10/11V, E*02:30V	E*02:11, E*02:30V
Rh29215	E*02:04, E*02:11, E*02:13V-short	E*02:11, E*02:13V-short

Supplementary Table 7. Genetic association analysis of *Mamu-E* variants with minor allele frequencies (MAF) > 0.1. Variants are clustered by correlations, with a threshold of 0.9. Clusters of size two without perfect correlations were split into individual clusters of size one (Methods). For each cluster, the positions of variants are shown along with their region (SP = signal peptide, TM = transmembrane domain, Cyto = cytoplasmic domain), type (snp = single nucleotide polymorphism, del = deletion), and MAF. Statistical tests for association were performed separately for clusters and individual variants, associating with protection outcome (protection) and vaccine group (group), with raw p-value and Benjamini-Hochberg adjusted p-values (FDR) shown.

Cluster	Number of variants	Protection		Group		Position	Region	Type	MAF	Protection		Group	
		p	FDR	p	FDR					p	FDR	p	FDR
1	21	0.755	1.000	0.948	1.000	126	Alpha1	snp	0.186	0.755	1.000	0.844	1.000
						197	Alpha1	snp	0.186	0.755	1.000	0.844	1.000
						283	Alpha1	snp	0.186	0.755	1.000	0.844	1.000
						962	TM	snp	0.203	0.755	1.000	0.948	1.000
						969	TM	snp	0.186	0.755	1.000	0.844	1.000
						996	TM	snp	0.186	0.755	1.000	0.844	1.000
						1047	Cyto	snp	0.203	0.755	1.000	0.948	1.000
						1048	Cyto	snp	0.203	0.755	1.000	0.948	1.000
						1050	Cyto	snp	0.203	0.755	1.000	0.948	1.000
						1079	Cyto	snp	0.203	0.755	1.000	0.948	1.000
						1098	3' UTR	snp	0.203	0.755	1.000	0.948	1.000
						1113	3' UTR	snp	0.203	0.755	1.000	0.948	1.000
						1115	3' UTR	snp	0.203	0.755	1.000	0.948	1.000
						1116	3' UTR	snp	0.203	0.755	1.000	0.948	1.000
						1151	3' UTR	snp	0.186	0.755	1.000	0.844	1.000
						1219	3' UTR	snp	0.212	0.755	1.000	0.948	1.000
						1243	3' UTR	snp	0.203	0.755	1.000	0.948	1.000
						1756	3' UTR	snp	0.203	0.755	1.000	0.948	1.000
						1907	3' UTR	snp	0.203	0.755	1.000	0.948	1.000
						2204	3' UTR	snp	0.186	0.755	1.000	0.844	1.000
						2394	3' UTR	snp	0.203	0.755	1.000	0.948	1.000
2	18	0.547	1.000	1.000	1.000	20	5' UTR	snp	0.280	0.547	1.000	1.000	1.000
						226	Alpha1	snp	0.263	0.547	1.000	1.000	1.000
						231	Alpha1	snp	0.263	0.547	1.000	1.000	1.000
						234	Alpha1	snp	0.263	0.547	1.000	1.000	1.000
						536	Alpha2	snp	0.314	1.000	1.000	0.939	1.000
						583	Alpha2	snp	0.305	0.763	1.000	0.863	1.000
						627	Alpha3	snp	0.314	0.364	1.000	0.983	1.000
						704	Alpha3	snp	0.280	0.547	1.000	1.000	1.000
						1029	Cyto	snp	0.280	0.547	1.000	1.000	1.000
						1062	Cyto	snp	0.280	0.547	1.000	1.000	1.000
						1093	3' UTR	snp	0.280	0.547	1.000	1.000	1.000
						1122	3' UTR	snp	0.280	0.547	1.000	1.000	1.000
						1209	3' UTR	snp	0.263	0.543	1.000	0.862	1.000
						1738	3' UTR	snp	0.280	0.547	1.000	1.000	1.000
						2172	3' UTR	snp	0.280	0.547	1.000	1.000	1.000
						2213	3' UTR	snp	0.280	0.547	1.000	1.000	1.000
						2269	3' UTR	snp	0.280	0.547	1.000	1.000	1.000
						2449	3' UTR	snp	0.280	0.547	1.000	1.000	1.000
3	4	0.116	0.497	0.156	0.953	966	TM	snp	0.339	0.215	1.000	0.027	1.000
						970	TM	del	0.364	0.116	1.000	0.156	1.000
						976	TM	snp	0.364	0.116	1.000	0.156	1.000
						977	TM	snp	0.364	0.116	1.000	0.156	1.000
4	3	1.000	1.000	0.840	1.000	205	Alpha1	snp	0.144	1.000	1.000	0.828	1.000
						645	Alpha3	snp	0.169	1.000	1.000	0.840	1.000
						984	TM	snp	0.169	1.000	1.000	0.840	1.000
5	2	0.281	0.842	0.663	1.000	5	5' UTR	snp	0.119	0.281	1.000	0.663	1.000
						940	TM	snp	0.119	0.281	1.000	0.663	1.000
6	1	0.537	1.000	0.238	0.953	390	Alpha2	snp	0.237	0.537	1.000	0.238	1.000
7	1	0.763	1.000	0.168	0.953	392	Alpha2	snp	0.305	0.763	1.000	0.168	1.000
8	1	0.124	0.497	0.855	1.000	623	Alpha2	snp	0.398	0.124	1.000	0.855	1.000
9	1	0.116	0.497	0.849	1.000	988	TM	snp	0.432	0.116	1.000	0.849	1.000
10	1	1.000	1.000	0.510	1.000	33	SP	snp	0.144	1.000	1.000	0.510	1.000
11	1	0.721	1.000	0.561	1.000	133	Alpha1	snp	0.127	0.721	1.000	0.561	1.000
12	1	1.000	1.000	0.339	1.000	185	Alpha1	snp	0.288	1.000	1.000	0.339	1.000

Supplementary Table 8. PCR primers used for long-range PCR of *Mamu-E* alleles.

Amplicon	Forward	Sequence	Reverse	Sequence
**Amp1**	mamuE-F2	TCAGGAGGTGAGAGCGCAGAACAATGTTCC	mamuE-R2	CACACAAGGCAGCTGTCTCAGGCTACAGAAG
**Amp2**	mamuE-F1	ACTGATTTACACAGTTTACACAGTCCCTG	mamuE-R3	ACAGTAAGTGATGACACTCTGAACAGCCCA
**Amp3**	mamuE-F3	TAACCTGGTCGTGTCCTTCTTCCTGGATAC	mamuE-R4	CCACAACCCAGTCCTGTCCCATCTGCCC
