# Supplementary material for: Alternative splicing and genetic variation of mhc-e: implications for rhesus cytomegalovirus-based vaccines
Source: Commun Biol. 2022 Dec 19;5:1387. doi: 10.1038/s42003-022-04344-2 (PMC9762870; doi:10.1038/s42003-022-04344-2)
Supplement: Supplementary file 2 — Description of Additional Supplementary Data [file 42003_2022_4344_MOESM2_ESM.docx]

**Description of Additional Supplementary Files**

**File name:** Supplementary Data 1

**Description:** The source data for Figures 3-5 and Supplementary Figures 1, 3-5, and 7-9
